# Switching HIV Treatment in Adults Based on CD4 Count Versus Viral Load Monitoring: A Randomized, Non-Inferiority Trial in Thailand

**DOI:** 10.1371/journal.pmed.1001494

**Published:** 2013-08-06

**Authors:** Gonzague Jourdain, Sophie Le Cœur, Nicole Ngo-Giang-Huong, Patrinee Traisathit, Tim R. Cressey, Federica Fregonese, Baptiste Leurent, Intira J. Collins, Malee Techapornroong, Sukit Banchongkit, Sudanee Buranabanjasatean, Guttiga Halue, Ampaipith Nilmanat, Nuananong Luekamlung, Virat Klinbuayaem, Apichat Chutanunta, Pacharee Kantipong, Chureeratana Bowonwatanuwong, Rittha Lertkoonalak, Prattana Leenasirimakul, Somboon Tansuphasawasdikul, Pensiriwan Sang-a-gad, Panita Pathipvanich, Srisuda Thongbuaban, Pakorn Wittayapraparat, Naree Eiamsirikit, Yuwadee Buranawanitchakorn, Naruepon Yutthakasemsunt, Narong Winiyakul, Luc Decker, Sylvaine Barbier, Suporn Koetsawang, Wasna Sirirungsi, Kenneth McIntosh, Sombat Thanprasertsuk, Marc Lallemant, PHPT-3 study team

**Affiliations:** 1 Unité Mixte Internationale 174, Institut de Recherche pour le Développement (IRD)-Programs for HIV Prevention and Treatment (PHPT), Chiang Mai, Thailand; 2 Department of Medical Technology, Faculty of Associated Medical Sciences, Chiang Mai University, Chiang Mai, Thailand; 3 Department of Immunology and Infectious Diseases, Harvard School of Public Health, Boston, Massachusetts, United States of America; 4 Unité Mixte de Recherche 196, Centre Français de la Population et du Développement, (INED-IRD-Paris V University), Paris, France; 5 Department of Statistics, Faculty of Science, Chiang Mai University, Chiang Mai, Thailand; 6 Prapokklao Hospital, Ministry of Public Health, Chanthaburi, Thailand; 7 Rayong Hospital, Ministry of Public Health, Rayong, Thailand; 8 Mae Chan Hospital, Ministry of Public Health, Chiang Rai, Thailand; 9 Phayao Provincial Hospital, Ministry of Public Health, Phayao, Thailand; 10 Hat Yai Hospital, Ministry of Public Health, Songkla, Thailand; 11 Lamphun Hospital, Ministry of Public Health, Lamphun, Thailand; 12 Sanpatong Hospital, Ministry of Public Health, Chiang Mai, Thailand; 13 Samutsakhon Hospital, Ministry of Public Health, Samutsakhon, Thailand; 14 Chiangrai Prachanukroh Hospital, Ministry of Public Health, Chiang Rai, Thailand; 15 Chonburi Hospital, Ministry of Public Health, Chonburi, Thailand; 16 Maharat Nakhon Ratchasima Hospital, Ministry of Public Health, Nakhon Ratchasima, Thailand; 17 Nakornping Hospital, Ministry of Public Health, Chiang Mai, Thailand; 18 Buddhachinaraj Hospital, Ministry of Public Health, Pitsanuloke, Thailand; 19 Ratchaburi Hospital, Ministry of Public Health, Ratchaburi, Thailand; 20 Lampang Hospital, Ministry of Public Health, Lampang, Thailand; 21 Mahasarakam Hospital, Ministry of Public Health, Mahasarakam, Thailand; 22 Bhuddasothorn Hospital, Ministry of Public Health, Chachoengsao, Thailand; 23 Samutprakarn Hospital, Ministry of Public Health, Samutprakarn, Thailand; 24 Chiang Kham Hospital, Ministry of Public Health, Phayao, Thailand; 25 Nong Khai Hospital, Ministry of Public Health, Nong Khai, Thailand; 26 Regional Health Promotion Centre 6, Ministry of Public Health, Khon Kaen, Thailand; 27 Family Health Research Center, Mahidol University, Bangkok, Thailand; 28 Children's Hospital, Department of Pediatrics, Harvard Medical School, Boston, Massachusetts, United States of America; 29 Ministry of Public Health, Nonthaburi, Thailand; St. Vincent's Hospital, Australia

## Abstract

**Background:**

Viral load (VL) is recommended for monitoring the response to highly active antiretroviral therapy (HAART) but is not routinely available in most low- and middle-income countries. The purpose of the study was to determine whether a CD4-based monitoring and switching strategy would provide a similar clinical outcome compared to the standard VL-based strategy in Thailand.

**Methods and Findings:**

The Programs for HIV Prevention and Treatment (PHPT-3) non-inferiority randomized clinical trial compared a treatment switching strategy based on CD4-only (CD4) monitoring versus viral-load (VL). Consenting participants were antiretroviral-naïve HIV-infected adults (CD4 count 50–250/mm^3^) initiating non-nucleotide reverse transcriptase inhibitor (NNRTI)-based therapy. Randomization, stratified by site (21 public hospitals), was performed centrally after enrollment. Clinicians were unaware of the VL values of patients randomized to the CD4 arm. Participants switched to second-line combination with confirmed CD4 decline >30% from peak (within 200 cells from baseline) in the CD4 arm, or confirmed VL >400 copies/ml in the VL arm. Primary endpoint was clinical failure at 3 years, defined as death, new AIDS-defining event, or CD4 <50 cells/mm^3^. The 3-year Kaplan-Meier cumulative risks of clinical failure were compared for non-inferiority with a margin of 7.4%. In the intent to treat analysis, data were censored at the date of death or at last visit. The secondary endpoints were difference in future-drug-option (FDO) score, a measure of resistance profiles, virologic and immunologic responses, and the safety and tolerance of HAART. 716 participants were randomized, 356 to VL monitoring and 360 to CD4 monitoring. At 3 years, 319 participants (90%) in VL and 326 (91%) in CD4 were alive and on follow-up. The cumulative risk of clinical failure was 8.0% (95% CI 5.6–11.4) in VL versus 7.4% (5.1–10.7) in CD4, and the upper-limit of the one-sided 95% CI of the difference was 3.4%, meeting the pre-determined non-inferiority criterion. Probability of switch for study criteria was 5.2% (3.2–8.4) in VL versus 7.5% (5.0–11.1) in CD4 (*p* = 0.097). Median time from treatment initiation to switch was 11.7 months (7.7–19.4) in VL and 24.7 months (15.9–35.0) in CD4 (*p* = 0.001). The median duration of viremia >400 copies/ml at switch was 7.2 months (5.8–8.0) in VL versus 15.8 months (8.5–20.4) in CD4 (*p* = 0.002). FDO scores were not significantly different at time of switch. No adverse events related to the monitoring strategy were reported.

**Conclusions:**

The 3-year rates of clinical failure and loss of treatment options did not differ between strategies although the longer-term consequences of CD4 monitoring would need to be investigated. These results provide reassurance to treatment programs currently based on CD4 monitoring as VL measurement becomes more affordable and feasible in resource-limited settings.

**Trial registration:**

ClinicalTrials.gov NCT00162682

*Please see later in the article for the Editors' Summary*

## Abstract 2

Using a randomized controlled trial, Marc Lallemant and colleagues ask if a CD4-based monitoring and treatment switching strategy provides a similar clinical outcome compared to the standard viral load-based strategy for adults with HIV in Thailand.

*Please see later in the article for the Editors' Summary*

## Editors' Summary

**Background:**

About 34 million people (most of them living in low-and middle-income countries) are currently infected with HIV, the virus that causes AIDS. HIV infection leads to the destruction of immune system cells (including CD4 cells, a type of white blood cell), leaving infected individuals susceptible to other infections. Early in the AIDS epidemic, most HIV-infected individuals died within 10 years of infection. Then, in 1996, highly active antiretroviral therapy (HAART)—combined drugs regimens that suppress viral replication and allow restoration of the immune system—became available. For people living in affluent countries, HIV/AIDS became a chronic condition but, because HAART was expensive, HIV/AIDS remained a fatal illness for people living in resource-limited countries. In 2003, the international community declared HIV/AIDS a global health emergency and, in 2006, it set the target of achieving universal global access to HAART by 2010. By the end of 2011, 8 million of the estimated 14.8 million people in need of HAART in low- and middle-income countries were receiving treatment.

**Why Was This Study Done?:**

At the time this trial was conceived, national and international recommendations were that HIV-positive individuals should start HAART when their CD4 count fell below 200 cells/mm^3^ and should have their CD4 count regularly monitored to optimize HAART. In 2013, the World Health Organization (WHO) recommendations were updated to promote expanded eligibility for HAART with a CD4 of 500 cells/mm^3^ or less for adults, adolescents, and older children although priority is given to individuals with CD4 count of 350 cells/mm^3^ or less. Because HIV often becomes resistant to first-line antiretroviral drugs, WHO also recommends that viral load—the amount of virus in the blood—should be monitored so that suspected treatment failures can be confirmed and patients switched to second-line drugs in a timely manner. This monitoring and switching strategy is widely used in resource-rich settings, but is still very difficult to implement for low- and middle-income countries where resources for monitoring are limited and access to costly second-line drugs is restricted. In this randomized non-inferiority trial, the researchers compare the performance of a CD4-based treatment monitoring and switching strategy with the standard viral load-based strategy among HIV-positive adults in Thailand. In a randomized trial, individuals are assigned different interventions by the play of chance and followed up to compare the effects of these interventions; a non-inferiority trial investigates whether one treatment is not worse than another.

**What Did the Researchers Do and Find?:**

The researchers assigned about 700 HIV-positive adults who were beginning HAART for the first time to have their CD4 count (CD4 arm) or their CD4 count and viral load (VL arm) determined every 3 months. Participants were switched to a second-line therapy if their CD4 count declined by more than 30% from their peak CD4 count (CD4 arm) or if a viral load of more than 400 copies/ml was recorded (VL arm). The 3-year cumulative risk of clinical failure (defined as death, a new AIDS-defining event, or a CD4 count of less than 50 cells/mm^3^) was 8% in the VL arm and 7.4% in the CD4 arm. This difference in clinical failure risk met the researchers' predefined criterion for non-inferiority. The probability of a treatment switch was similar in the two arms, but the average time from treatment initiation to treatment switch and the average duration of a high viral load after treatment switch were both longer in the CD4 arm than in the VL arm. Finally, the future-drug-option score, a measure of viral drug resistance profiles, was similar in the two arms at the time of treatment switch.

**What Do These Findings Mean?:**

These findings suggest that, in Thailand, a CD4 switching strategy is non-inferior in terms of clinical outcomes among HIV-positive adults 3 years after beginning HAART when compared to the recommended viral load-based switching strategy and that there is no difference between the strategies in terms of viral suppression and immune restoration after 3-years follow-up. Importantly, however, even though patients in the CD4 arm spent longer with a high viral load than patients in the VL arm, the emergence of HIV mutants resistant to antiretroviral drugs was similar in the two arms. Although these findings provide no information about the long-term outcomes of the two monitoring strategies and may not be generalizable to routine care settings, they nevertheless provide reassurance that using CD4 counts alone to monitor HAART in HIV treatment programs in resource-limited settings is an appropriate strategy to use as viral load measurement becomes more affordable and feasible in these settings.

**Additional Information:**

Please access these websites via the online version of this summary at http://dx.doi.org/10.1371/journal.pmed.1001494.

- The World Health Organization provides information on all aspects of HIV/AIDS (in several languages); its 2010 recommendations for antiretroviral therapy for HIV infection in adults and adolescents are available as well as the June 2013 Consolidated guidelines on the use of antiretroviral drugs for treating and preventing HIV infection: recommendations for a public health approach
- The 2012 UNAIDS World AIDS Day Report provides up-to-date information about the AIDS epidemic and efforts to halt it
- Information is available from the US National Institute of Allergy and Infectious Diseases on HIV infection and AIDS
- NAM/aidsmap provides basic information about HIV/AIDS and summaries of recent research findings on HIV care and treatment
- Information is available from Avert, an international AIDS charity on many aspects of HIV/AIDS, including information on the global HIV/AIDS epidemic, on HIV and AIDS in Thailand, on universal access to AIDS treatment, and on starting, monitoring and switching HIV treatment (in English and Spanish)
- The UK National Health Service Choices website provides information (including personal stories) about HIV and AIDS
- More information about this trial (the PHPT-3 trial) is available
- Patient stories about living with HIV/AIDS are available through Avert; the nonprofit website Healthtalkonline also provides personal stories about living with HIV, including stories about HIV treatment

## Introduction

Since the mid 1990s, highly active antiretroviral therapy (HAART) has radically modified AIDS prognosis by suppressing viral replication and thus allowing immune restoration [1]–[3]. Maximal and durable viral suppression is expected to impede the development of drug resistance and to lead to the restoration of immunological function, improvement of quality of life, and reduction of HIV-related morbidity, mortality, and transmission. Monitoring of viral load (VL) is central to this therapeutic approach and to national guidelines in most resource-rich settings and was recently recommended as part of the WHO 2013 consolidated guidelines [4],[5].

However, in low- and middle-income countries, with limited resources and restricted access to more costly second and third-line drugs, the utility of this approach is debated [6]–[8]. Moreover, a VL-based monitoring strategy may lead to more frequent treatment changes, limiting future drug options. Three randomized trials compared clinical monitoring with clinical-plus-laboratory monitoring in adult patients in sub-Saharan Africa [9]–[11], but none of them directly compared CD4-monitoring versus CD4 plus VL monitoring. We therefore designed this study to determine whether, in therapy-naïve patients, monitoring by VL is optimal for therapeutic decision making, or whether a CD4-based strategy would lead to non-inferior clinical outcomes. The purpose of the study was to test the non-inferiority of a CD4-based monitoring and switching strategy compared to the standard VL-based monitoring and switching strategy among antiretroviral (ARV)-naive adults treated with non-nucleotide reverse transcriptase inhibitor (NNRTI)-containing regimens in Thailand.

## Methods

### Trial Design

This was a multicenter, randomized, non-inferiority trial conducted in 21 public hospitals throughout Thailand (ClinicalTrials.gov NCT00162682). The primary objective was to compare the 3-y clinical outcomes of HIV-infected adults initiating HAART, followed according to a monitoring-switching strategy either based on CD4 cell count (CD4), or on VL. The secondary objectives were (i) to compare future ARV treatment options, taking into account the profile of resistance mutations; (ii) to assess virologic and immunologic responses by arm; and (iii) to evaluate the safety and tolerance of HAART.

### Participants

Confirmed HIV-infected patients, 18 y or older, were eligible if they could be followed at a study site and provided written informed consent. Inclusion criteria included confirmed CD4-cell count of 50–250 cells/mm^3^ and absence of prior ARV therapy (except zidovudine during pregnancy or nevirapine during labor). Exclusion criteria included pregnancy, opportunistic infection or medical condition interfering with study participation, hepatitis B or C co-infection, or any of the following: hemoglobin <8.0 g/dl, neutrophil count <1,000 cells/mm^3^, alanine transaminase (ALT), aspartate aminotransferase (AST), or total bilirubin >5.0× upper limit of normal (ULN), serum creatinine >1.0× ULN, platelet count <50,000/mm^3^, pancreatic amylase >2.0× ULN, or total amylase >2.0× ULN plus symptoms of pancreatitis.

### Randomization and Switching Criteria

After treatment initiation, participants were randomly assigned in blocks of four, stratified by site and CD4 level (±100 cells/mm^3^), to one of two monitoring-switching strategies: (1) VL arm: switch if confirmed decreased <1 log at 3 mo or confirmed VL above 400 copies/ml thereafter; (2) CD4 arm: switch if confirmed CD4 count declined >30% from peak value (defined as the highest average of two consecutive CD4 counts) unless CD4 remained >200 cells above baseline. Using a pseudorandom number generator (Mersenne twister), a statistician produced the randomization lists and encrypted them in a database before the initiation of the study. Only the study statisticians were allowed to access the randomization lists to maintain blinding of other research staff. Randomization was performed centrally at the study coordination center in Chiang Mai, by a research assistant. The arm assigned to each patient was disclosed to the site physician after randomization. Even though blinding was not feasible, clinicians who were responsible for enrolling and following-up with study participants were unaware of the VL values of patients randomized to the CD4 arm.

### Follow-up

Patients were seen 2 wk after ARV initiation for a medical examination and blood chemistry evaluation, to ensure adherence and detect early toxicities. Patients were then seen monthly for clinical evaluation, adherence assessment by pill count and self-report questionnaire, safety and tolerance evaluation, and drug refill. Cotrimoxazole and fluconazole prophylaxis was provided per WHO guidelines [12]. Hematology, ALT, CD4 count, and pregnancy tests and VL were performed at enrollment and then every 3 mo. Creatinine, bilirubin, AST, glucose, triglycerides, cholesterol, and amylase were measured every 6 mo. In case of intolerance to one drug, that drug was replaced. Serious adverse events (as defined by the International Conference on Harmonization, Good Clinical Practices [ICH GCP]) were reported to the Ministry of Public Health. Adverse event grading was based on the Division of AIDS, NIAID Table [13]. Patients were monitored according to protocol until the last enrollee had been on study for 3 y.

### Laboratory Assessments

Plasma HIV RNA was measured using the Cobas Amplicor HIV-1 Monitor kit (version 1.5, Roche Molecular Systems). HIV genotypic resistance was performed retrospectively for all patients who met the per-protocol switching criteria and had detectable VL on the last sample available before switch using ViroSeq HIV-1 Genotyping system version 2.0 (Applied Biosystems). Both were performed at the Faculty of Associated Medical Sciences, Chiang-Mai University and quality assured by the Virology Quality Assurance Proficiency Program (VQA). CD4 counts were measured using a flow cytometer at each hospital laboratory with quality control from the Center of Excellence for Flow Cytometry, Mahidol University, Bangkok, Thailand and the United Kingdom National External Quality Assessment Service (UKNEQAS). Resistance mutations were identified using the 2009 International AIDS Society (US) tables. Each mutation was assigned a penalty score derived from the Stanford HIVdb Sequence Analysis Programs (version 6.0.8) and ARV drug resistance was inferred by adding the penalty scores of each mutation.

### Antiretroviral Treatment

Upon enrollment, participants initiated a regimen containing nevirapine or efavirenz, plus lamivudine with stavudine or zidovudine. From April 2006, tenofovir plus emtricitabine (Truvada) became available and was widely used in combination with efavirenz. When switching criteria were reached, a protease inhibitor (PI)-based regimen, usually indinavir/ritonavir or lopinavir/ritonavir, depending on availability, was provided. Before treatment switches, causes for viral rebound or immunological deterioration were investigated, with attention to adherence, toxicities, and co-infections. Drug changes within class or between classes for reasons of toxicity were not considered “protocol switches” in the analysis.

### Outcomes

The primary endpoint was clinical failure defined as confirmed CD4 <50 cells/mm^3^, first or new AIDS-defining event, or death. An independent committee reviewed and classified all AIDS-defining events. The main secondary endpoint was the number of drugs remaining available for treatment at the time of switch (future drug options, denoted FDO), calculated from resistance mutations [14]. Two FDO scores were calculated: FDO-1 based on the number of drug classes with one or more drugs to which the virus was susceptible (NC) with extra credit (0.3) for full susceptibility in NRTI or PI classes; and FDO-2 calculated as NC + the number of drugs to which the virus was susceptible (ND) divided by the total number (19) of drugs available +1, i.e., NC + (ND/20). Other secondary endpoints were the rate of switch to second-line regimens per protocol criteria, virologic response (percent of subjects below 50 copies/ml at 3 y), immunological status (CD4 cell count at 3 y), and serious adverse events.

### Sample Size

On the basis of a literature review at the time the study was planned, the Kaplan-Meier cumulative 3-y risk of clinical failure on VL monitoring was expected to be 5% per year, or 14% over 3 y [15]–[18]. For the primary analysis, a noninferiority margin (delta) of 7.4%, corresponding to a hazard ratio of 1.6, was considered acceptable in view of the expected benefit of the CD4 monitoring strategy. Using a one-sided confidence interval (CI) approach, a sample size of 304 evaluable patients per arm ensured 80% power to rule out a difference greater than delta. Assuming two interim analyses and 15% unevaluable, 350 patients per arm were required.

### Statistical Methods

The primary analysis compared the CD4 versus VL arm based on the Kaplan-Meier estimates of clinical/immunologic failure at 36 mo. All randomized patients were included in the final intent to treat analysis. Participants who died, withdrew from the study, or were lost to follow-up (defined as those who missed all visits for over 6 mo and no contact) were included and data were censored at date of death or at last visit. Participants who completed the study schedule were censored on April 1, 2010. Distributions were compared using the Fisher exact test and Wilcoxon rank-sum test. Additional analyses studied baseline factors associated with clinical failure using Log rank tests and Cox proportional hazards models after testing that the Cox proportional hazards model assumptions were met (covariate effects not changing over time, and flat slope in the regression of time versus residuals).

### Study Monitoring

In addition to the Data and Safety Monitoring Board (DSMB), a Resistance Experts Committee was constituted to provide expertise in support of the DSMB regarding resistance mutations and their clinical implications. Outcomes, safety and resistance profiles by arm, were presented during the two interim analyses.

The study protocol was approved by the Ethical Committees of the Thai Ministry of Public Health, Chiang Mai University Faculty of Associated Medical Sciences, Harvard School of Public Health Institutional Review Board, and local hospitals when applicable.

## Results

**Figure 1:**
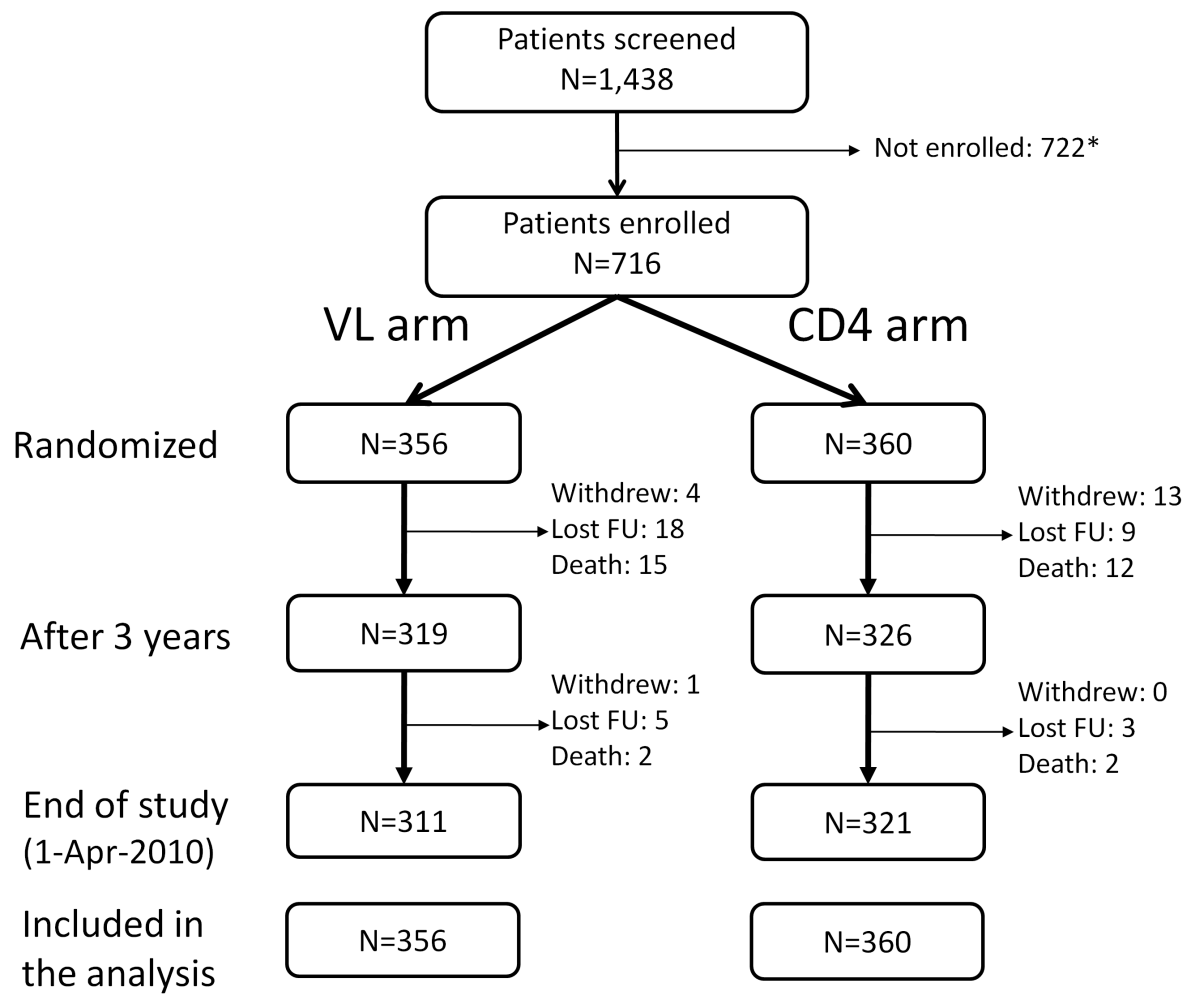
Patients' disposition. All patients randomized were included in the final analysis with patients who were lost to follow-up (FU), withdrew, or died considered as censored when last seen or at the time of death. *Reasons for not being enrolled: 158 subjects with CD4 <50 or >250 cells/mm^3^; 284 drop-out before enrollment (no information available for more than 2 mo); 101 with hepatitis B or C infection; 179 with other exclusion criteria such as pregnancy, opportunistic infections, or laboratory values outside the required ranges. 18 patients withdrew from the study: 11 because they moved to another region, five because they found the follow-up too frequent, and two because of side effects of the treatments.

From May 2005 to April 2007, 716 participants were recruited and randomized: 356 to the VL arm and 360 to the CD4 arm (Figure 1). All participants were included in the analysis. Baseline characteristics are provided in Table 1. The two arms were balanced with respect to all baseline characteristics except sex (58% female in the VL arm and 66% in the CD4 arm, *p* = 0.03). Initial regimens were efavirenz-based in 65% and nevirapine-based in 35% of participants; 66% were in combination with tenofovir-emtricitabine and 34% with zidovudine or stavudine plus lamivudine.

**Table 1:** Characteristics of the patients at baseline, according to randomization arm.

Study Arm	VL Arm	CD4 Arm	Total
Total randomized patients^a^	356	360	716
Sex: Females (%)	207 (58%)	239 (66%)	446 (62%)
Median age – y (IQR)	35.4 (31.0–41.2)	36.2 (31.4–41.4)	35.7 (31.2–41.2)
Median BMI (IQR)	20.7 (18.8–23.0)	20.9 (19.0–22.9)	20.8 (19.0–23.0)
Median absolute CD4 count – cells/mm^3^ (IQR)	146 (90–201)	144 (90–198)	144 (90–199)
Median VL at enrolment – log_10_ copies per ml (IQR)	4.9 (4.3–5.2)	4.8 (4.3–5.2)	4.8 (4.3–5.2)
CDC stage – *n* (%)			
B	83 (23%)	90 (25%)	173 (24%)
C	74 (21%)	64 (18%)	138 (19%)
First-line regimen – *n* (%)			
Nevirapine-based	119 (33.4%)	131 (36.4%)	250 (35.0%)
Efavirenz-based	236 (66.3%)	229 (63.6%)	465 (64.9%)
Includes tenofovir	235 (66.0%)	238 (66.1%)	473 (66.1%)
Laboratory – median (IQR)			
Hemoglobin – g/dl	12.0 (10.9–13.3)	11.8 (10.7–13.0)	12.0 (10.8 13.1)
Alanine aminotransferase – U/L	28.0 (17.5–42.0)	28.0 (18.0–40.0)	28.0 (18.0–41.5)
Creatinine – mg/dl	0.8 (0.7–1.0)	0.8 (0.7–0.9)	0.8 (0.7–0.9)
Total bilirubin — mg/dl	0.5 (0.4–0.7)	0.5 (0.4–0.6)	0.5 (0.4–0.6)
Triglycerides —mg/dl	129 (95–182)	130.0 (95–183)	130 (95–183)
Cholesterol — mg/dl	158 (135–180)	157 (136–184)	158 (135–183)

a There were eight protocol deviations reported related to inclusion criteria: One patient was not ARV naive, one woman was pregnant, one was chronically infected with hepatitis C, two had hemoglobin level <8.0 g/dl, two had absolute neutrophil count <1,000 cells/mm^3^, one had serum creatinine above 1.0× ULN.

The study was completed and unblinded on April 1, 2010, after the last-enrolled patient reached 3 y of follow-up. The DSMB recommended pursuit of the study as planned at the two interim analyses. At 3 y following enrollment, 27 patients had died, 27 were lost-to-follow up, and 18 had withdrawn (Figure 1), with 319 participants (90%) in VL and 326 (91%) in CD4 alive and on follow-up. There was no statistically significant difference between arms in terms of loss to follow-up or withdrawal (*p* = 0.63). At the end of study, after a median follow-up of 43.5 mo (interquartile range [IQR] 38.8–48.4), retention remained high (93.7%); the ARV regimens were PI-based in 14% of the participants (indinavir/ritonavir in 62%, lopinavir/ritonavir in 23%, saquinavir/ritonavir in 13%, and atanazavir/ritonavir in 2%), efavirenz-based in 64%, and nevirapine-based in 22% of participants; 76% were in combination with tenofovir-emtricitabine or -lamivudine.

Compliance to scheduled visits and adherence to therapy were both excellent with no difference between arms—4.2% of patients' visits (213 among a total of 5,020 patients' visits) with >15 d difference between scheduled and actual dates, and 7.3% (52/716) reporting >1 missed dose/week. Seventy-two patients experienced treatment interruption (64 patients one episode, six patients two, and two patients three). The reasons for the first interruptions were intolerance or toxicity in 48 cases (67%), self-interruption or missed visits in 22 cases (31%), and surgery in two cases. The median duration of the first interruption was 3.8 wk (IQR 1.3–8.6) with no difference between arms.

### Primary Outcome: Clinical Failure

Overall, 58 patients (30 in the VL arm and 28 in the CD4 arm) reached the primary endpoint of clinical failure: three experienced a CD4 count decrease below 50 cells/mm^3^, 33 developed a new AIDS-defining event (including nine followed by death), and 22 died (Table 2). Nineteen of the 26 AIDS-defining events that occurred in the first 6 mo were classified as possibly related to immune reconstitution. The AIDS-defining events included 13 tuberculosis or mycobacterium infections (four fatal), seven cryptococcal diseases (one fatal), five sepsis (four fatal), four *Pneumocystis jerovicii* pneumonia, and four systemic *Penicillium marneffei* infections.

**Table 2:** Number of primary outcomes by arm.

First Clinical Failures	VL	CD4	Total
AIDS-defining events^a^	18^b^	15^c^	**33** a
Deaths	11^d^	11^e^	**22**
CD4 count <50 cells/mm^3^	1	2	**3**
**Total**	**30**	**28**	**58**

a Including nine cases followed by death.

b Tuberculosis (8), cryptococcal meningitis (3), *P. jirovecii* pneumonia (2), systemic *P. marneffei* (2), disseminated *Mycobacterium avium intracellulare* (1), sepsis (2).

c Tuberculosis (4), cryptococcal meningitis (4), *P. jirovecii* pneumonia (2), systemic *P. marneffei* (2), sepsis (3).

d Sepsis (3), cerebrovascular accidents (2), heart failure (1), asthma attack (1), *P. jirovecii* pneumonia (1), hepatic failure (1), unknown cause (2).

e Heart failure (3), cancer (2, 1 breast cancer, 1 liver cancer), suicide (2), renal failure (1), hepatic failure (1), pneumonia (1), sepsis (1).

**Figure 2:**
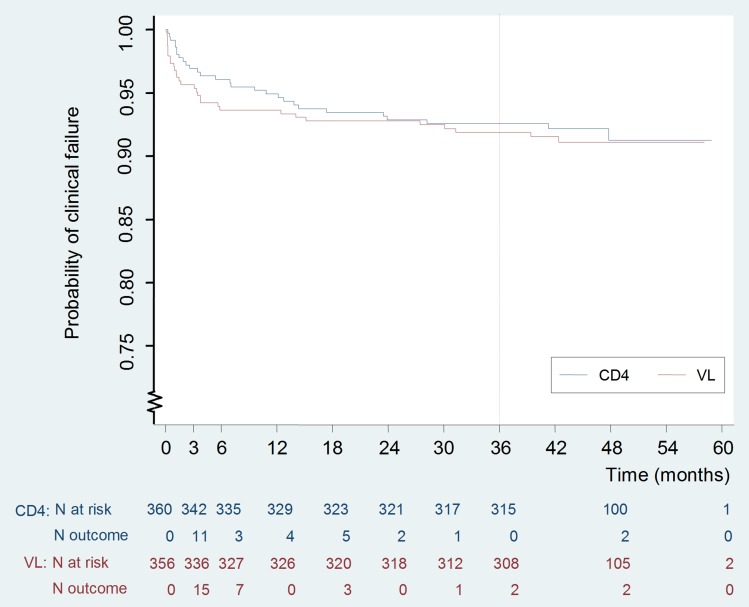
Kaplan Meier curve of occurrence of clinical failure.

The Kaplan-Meier risk of clinical failure at 3-y was not significantly different between the two arms: 8.0% (95% CI 5.6–11.4) in the VL versus 7.4% (5.1–10.7) in the CD4 arm (*p* = 0.74) (Figure 2; Table 3). The 95% CI of the difference, −0.6%, was −4.5% to 3.4%. The upper limit of this CI was below the pre-determined criterion for non-inferiority, 7.4%. The corresponding hazard ratio was 0.92, and its 95% CI was 0.55–1.53, also within the preset hazard ratio non-inferiority limit of 1.6. Nearly half of the failures occurred during the first 3 mo of therapy (15/30 and 11/28 in the VL and CD4 arms, respectively) before the tested monitoring strategies could have any clinical impact. At 3-y, the cumulative risk of death was 4.3% (2.6–7.1) versus 3.4% (2.0–6.0), respectively (*p* = 0.57).

**Table 3:** Primary outcomes (clinical failure) at 3 y: death, new AIDS-defining events, or confirmed CD4 <50/mm^3^.

Clinical Failure (Death, New AIDS-Defining Event, CD4 <50/mm^3^)	VL*n* = 356	CD4*n* = 360	*p*-Value
Kaplan Meier Risk (95% CI)	8.0% (5.6–11.4)	7.4% (5.1–10.7)^a^	0.74
Rate per 100 patient years (95% CI)	2.5 (1.8–3.6)	2.3 (1.6–3.4)	0.76
**Death**			
Kaplan Meier risk (95% CI)	4.3% (2.6–7.1)	3.4% (2.0–6.0)	0.57
Mortality rate per 100 patient years (95% CI)	1.4 (0.8–2.2)	1.1 (0.7–1.9)	0.74

a The 95% CI of the difference, −0.6% was −4.5% to 3.4%. The upper limit of this CI was below the pre-determined criterion for non-inferiority, 7.4%.

Baseline factors associated with clinical failure by univariate analysis included US Centers for Disease Control and Prevention (CDC) stage B or C, anemia (hemoglobin ≤10 g/dl), CD4 count below median, i.e., 150 cells/mm^3^ (all *p*<0.001), VL above 5 log_10_ copies/ml (*p* = 0.001), and body mass index (BMI) below 18.5 (*p* = 0.002) (Table 4). Age, sex, initial ARV regimen, and switching strategy were not associated with the primary outcome. Upon multivariable analysis, factors independently associated with clinical failure were baseline anemia (*p* = 0.001), CDC stage B or C (*p* = 0.002), and low CD4 count (*p* = 0.04).

**Table 4:** Risk factors for clinical failure.

Characteristics	Univariable Analysis			Multivariable Analysis		
	HR^a^	95% CI	*p*-Value	AHR^b^	95% CI	*p*-Value
Hemoglobin ≤10 g/dl	3.7	2.2–6.5	<0.001	2.8	1.5–5.0	0.001
CDC stage B or C	3.9	2.2–7.0	<0.001	2.6	1.4–4.9	0.002
CD4 count <150 cells/mm^3^	2.9	1.6–5.4	<0.001	1.9	1.0–3.5	0.04
VL >5 log_10_ copies/ml	2.4	1.4–4.1	0.001	1.4	0.9–2.6	0.16
BMI <18.5	2.3	1.4–4.0	0.002	1.5	0.8–2.6	0.17
Female sex	0.8	0.4–1.30	0.28	0.8	0.5–1.4	0.39
Randomization arm (VL)	1.1	0.7–1.8	0.74	1.1	0.6–1.8	0.84

a HR, hazard ratio.

b Adjusted HR (AHR, Cox proportional analysis) adjusted for hemoglobin, CDC stage, CD4 count, VL, BMI, sex, and randomization arm.

### Secondary Outcomes

#### Switch for study criteria

Fifty of the 716 enrolled patients (14%) were switched to second-line regimens as per protocol criteria (19 in the VL arm and 31 in the CD4 arm). The 36-mo probabilities of switch for protocol were not significantly different, 5.2% (3.2–8.4) in VL versus 7.5% (5.0–11.1) in CD4, *p* = 0.10. However, the median time from enrollment to switch was significantly shorter in the VL arm (11.7 versus 24.7 mo, *p* = 0.001) (Table 5).

**Table 5:** Treatment switch to second-line, PI-based treatment.

Characteristics	VL*n* = 356	CD4*n* = 360	*p*-Value
Probability of switch for study criteria (Kaplan-Meier estimate, 95% CI)	5.2% (3.2–8.4)	7.5% (5.0–11.1)	0.10
Median time to switch – mo (IQR)	11.7 (7.7–19.4)	24.7 (15.9–35.0)	0.001
Median CD4 at switch –cells/mm^3^ (IQR)	246 (158–323)	196 (144–347)	0.62
Median VL at switch excluding patients with VL <400 copies/ml – log_10_ copies/ml (IQR)	3.8 (3.2–4.3)	3.9 (3.2–4.4)	0.96
Median duration with viral replication (>400 copies/ml) before switch– mo (IQR)^a^	7.2 (5.8–8.0)	15.8 (8.5–20.4)	0.002
Median duration with viral replication (>50 copies/ml) before switch– mo (IQR)^b^	7.7 (6.0–13.7)	17.2 (9.7–23.9)	<0.001

a Calculated starting in the middle of the time period between the last VL <400 copies/ml and first VL above.

b Calculated starting in the middle of the time period between the last VL <50 copies/ml and first VL above.

Among the 31 patients who switched to a second-line regimen for protocol criteria in the CD4 arm, 15 had VL <400 copies/ml at time of switch. In six of these cases the CD4 count subsequently increased, in seven it remained stable, and in two it fell further. Of the 19 patients who switched for criteria in the VL arm, four would have switched earlier if the CD4 criteria had been used.

At the time of switch, median CD4 count and VL among those with VL >400 copies/ml were not different between arms (Table 5). However, patients had a significantly shorter median duration of viremia >400 copies/ml in the VL arm than in the CD4 arm. At the 50 copies/ml threshold, the difference was even greater.

### Resistance and Future Drug Options

In the VL arm (Table 6), 18 of the 19 participants reaching switching criteria had interpretable genotyping results: 15 had at least one resistance mutation to NNRTI and 13 to NRTI, of whom two had at least three NRTI resistance mutations. In the CD4 arm (Table 7), all of the 16 participants reaching switching criteria with detectable VL had at least one resistance mutation to NNRTI and 13 to NRTI, of whom six had at least three NRTI resistance mutations. Thymidine analogue mutations (TAMs) were observed in two patients, but both had already one major NNRTI resistance mutation (Y181C) at baseline.

**Table 6:** Resistance mutations found at first ARV switch (VL arm).

Duration of Replication before Genotype^a^ (mo)	VL at Genotype (log_10_ Copies/ml)	NNRTI Resistance Mutations	NRTI Resistance Mutations
2^b^	4.63		
2	2.85	103N, 108I, 181C	184V
2.5	2.95		
2.5	3.8	181C	219E
3^b^	4.41	103N	
3.5	3.31		184V
3.5	3.85	106M, 227L	67N, 70E, 184V
4	4.16	103N, 181C	65R
4	4.34	101E, 181I	115F, 184V
5.5	5.87	98G, 181C, 190A	65R, 184V
5.5	4.1	103N, 106A, 190A	184V
6	2.81	108I, 181C	75M, 184V
6.5	3.84	101E, 190A, 318F	184V
7.5	4.98	103N	
8	4.42	181C	65R, 115F, 184V
9.5	3.58	188L	184V
10	3.88	190A	184V
11	3.88	103N	

This table shows 18 patients randomized to the VL arm who reached switching criteria of >400 copies/ml. The last samples collected before switch were genotyped. One participant with extensive PI resistance in the pretreatment specimen is omitted from this table.

a “Duration of replication before genotype” is defined as time from first detection of VL >400 copies/ml to genotyping. This may be shorter than the duration before ARV drug switching.

b These two participants were not included in calculations related to ARV drug switches since, although they met VL criteria for switching during the study, they both switched after the end of the study (April 1, 2010).

**Table 7:** Resistance mutations found at first ARV switch (CD4 arm).

Duration of Replication before Genotype^a^ (mo)	VL at Genotype (log_10_ Copies/ml)	NNRTI Resistance Mutations	NRTI Resistance Mutations
5.5	3.28	101E, 190A	184V
6.5	3.18	103N	
10	3.44	101P, 101Q, 106A, 103N, 225H	184V
10	2.82	103N	
10.5	3.32	103N, 225H	184V
10.5	4.05	108I, 181C	65R, 115F, 184V
10.5	4.4	98G, 103N, 225H	74I, 184V
13.5	4.09	101E, 179F, 181C	65R, 69S, 219R
13.5	4.23	103N	
16	3.72	101E, 181C, 230L	65R, 184I, 184V
20.5	3.65	103N	70N, 74I, 75M, 184V
21.5	3.24	98G, 101E, 103N, 190A	184V
24.5	3.9	101E, 181C	70R, 184V, 215V, 215I, 219Q^b^
24.5	4.5	101E, 181C	41L, 67N, 184V, 210W, 215Y^b^
26.5	3.2	103N, 108I	74I, 184V
27.5	5.13	103N, 190A	184V

This table includes all 16 participants in the CD4 arm who had VL >400 copies/ml at the time of switching.

a Duration of replication before measurement of genotype may be shorter than duration before ARV drug switching.

b These two participants both had Y181C mutations at baseline and were the only two of the participants switching to second-line regimens with major baseline NNRTI resistance mutations.

FDO scores were comparable in the two study arms (Table 8). At the time of switching, FDO-1 and FDO-2 scores were slightly higher in the VL arm than in the CD4 arm. Scores at the end of the study were similar to those at the time of switch.

**Table 8:** Mean, 95% CI, median, and IQR of the Future Drug Options scores, by trial arm.

FDO^a^	At Time of Switch			At the End of the Study		
	VL*n = *16	CD4*n = *15	*p*-Value^b^	VL*n = *18	CD4*n = *16	*p*-Value^b^
**FDO-1** c						
Mean	3.25	3.14	—	3.27	3.11	—
95% CI	3.04–3.46	2.89–3.39	—	3.08–3.47	2.84–3.37	—
Median	3.30	3.30	0.38	3.30	3.30	0.30
IQR	3.30–3.45	3.30–3.30	—	3.30–3.60	2.80–3.30	—
**FDO-2** d						
Mean	3.58	3.41	—	3.59	3.37	—
95% CI	3.35–3.80	3.15–3.67	—	3.39–3.79	3.09–3.65	—
Median	3.78	3.60	0.11	3.70	3.60	0.10
IQR	3.55–3.80	3.50–3.70	—	3.55–3.80	3.08–3.70	—

a FDO calculated using the following ARV drugs: nevirapine, efavirenz, delavirdine, etravirine; abacavir, didanosine, emtricitabine/lamivudine, stavudine, tenofovir, zidovudine; nelfinavir, indinavir, ritonavir, lopinavir. saquinavir, atazanavir, fosamprenavir, darunavir, tipranavir.

b *p*-Value from Wilcoxon Mann-Whitney test.

c FDO score 1: FDO-1 is calculated as the number of drug classes with one or more drug to which the virus was susceptible (NC) with extra credit (0.3) for full susceptibility in NRTI or PI classes.

d FDO score 2: FDO-2 is calculated as NC + the number of drugs to which the virus was susceptible (ND) divided by the total number (19) of drugs available + 1, i.e., NC+(ND/20).

### Virologic and Immunologic Responses at 3 y

At 3 y, the percentages of participants with VL <50 copies/ml were very high in both arms: 98.4% in the VL and 98.2% in CD4 arm. Similarly, the median CD4 cell counts at 3 y were high in both arms: 420 cells/mm^3^ (IQR 321–527) in VL and 426 (IQR 335–538) in the CD4 arm.

### Toxicity and Safety

There were 335 serious adverse events reported during the study among 198 patients, with no difference between arms: 112 (33.4%) were possibly or probably related to HIV and 67 (20.0%) to ARV toxicity (Table 9). At 3 y, the probability of occurrence of a serious event was 31.8% in the VL-s arm versus 29.5% in the CD4-s arm (*p* = 0.76). A total of 151 grade 3 adverse events were observed in 92 participants (73 in 45 patients in the VL arm and 78 in 47 patients in the CD4 arm). One hundred and twenty seven grade 4 adverse events were reported, 56 in 52 patients in the VL arm and 72 in 57 patients in the CD4 arm. Fifty patients (19 in the VL and 31 in the CD4 arm) changed treatment from NNRTI-based to PI-based regimens because of toxicities, most of which were rashes (11 cases in the VL arm and ten in the CD4 arm) or hepatic enzyme abnormalities (12 cases in the VL arm and seven in the CD4 arm).

**Table 9:** Serious adverse events by trial arm.

Serious Adverse Event	VL	CD4	Total
**Relationship with HIV**			
Definitively not related	79 (51.3%)	98 (54.1%)	177 (52.8%)
Probably not related	18 (11.7%)	28 (15.5%)	46 (13.7%)
Possibly related	15 (9.7%)	19 (10.5%)	34 (10.1%)
Probably related	14 (9.1%)	20 (11.0%)	34 (10.1%)
Definitively related	28 (18.2%)	16 (8.8%)	44 (13.1%)
**Total**	**154**	**181**	**335**
**Relationship with ARV**			
Definitively not related	100 (64.9%)	117 (64.6%)	217 (64.8%)
Probably not related	10 (6.5%)	29 (16.0%)	39 (11.6%)
Possibly related	16 (10.4%)	20 (11.0%)	36 (10.7%)
Probably related	20 (13.0%)	11 (6.1%)	31 (9.3%)
Unknown	8 (5.2%)	4 (2.2%)	12 (3.6%)
**Total**	**154**	**181**	**335**

## Discussion

In this randomized clinical trial of NNRTI-based HAART in an HIV-infected treatment-naïve population with 50 to 250 CD4/mm^3^, a CD4-based switching strategy was non-inferior in terms of clinical outcomes at 3 y of follow-up, compared to a reference VL-based switching strategy. Moreover, at study end there were no differences in terms of viral suppression and immune restoration between arms. Although the duration of detectable viral replication was longer in the CD4 arm, resistance testing showed similar profiles in the two arms.

Three other randomized monitoring trials conducted in sub-Saharan Africa have been published [9]–[11]. In all three the primary question was whether outcomes with clinical monitoring were as good as with clinical-plus-laboratory monitoring, using CD4 cells alone [11], or VL plus CD4 [9]. All three concluded that laboratory monitoring gave a better outcome. Only one study, the HBAC trial in Uganda, directly compared outcomes of CD4- versus viral-load–based monitoring, as part of their secondary analysis [16]. Participants in that study had more advanced disease at entry than in our study, with higher risk of death and new AIDS defining events, and no difference in outcomes at 3 y of follow-up was also observed. A preliminary report from a cluster randomized trial in Zambia comparing the effect of routine VL testing to the standard of care in which VL is used sparingly to adjudicate discrepancies between CD4 and clinical assessments, showed that VL monitoring did not reduce mortality over the first 36 mo of ART but resulted in earlier regimen change [19]. One international data-base analysis found earlier failure or death in Zambia and Malawi where patients were monitored with CD4 only, in comparison to South Africa where VL measurements were available [20]. This result may also reflect differences in health care environment.

The health consequences of the longer duration of viral replication in the CD4 arm of the study are difficult to gauge. The exploratory AIDS Clinical Trials Group Study A5115 that compared switching at high versus low VL thresholds in a population with median CD4 concentration of 421 cells/mm^3^ also found no differences in total or activated CD4 cells or FDO scores at study end [21],[22]. The SMART treatment interruption study raised concerns, subsequently confirmed [23], that viral replication leads to more rapid immunologic deterioration and immune activation, increasing the risk of cardiovascular events, cancer, and hepatic dysfunction [24],[25]. No excess of these events was observed in the CD4 arm in our study, but the numbers were not large, ARV drugs were not discontinued with likely consequent partial control of viral replication, and CD4 cell numbers were preserved during viremia.

Although more switches were expected in the VL than in the CD4 arm, the opposite was observed. In the CD4 arm, 31 patients switched for study criteria but almost half of them (15) had VL <400 copies/ml at the time of switch. It is well known that a drop in CD4 cells does not necessarily correlate with virological failure [26]–[28], and while these switches might be viewed as unnecessary, they did not appear to do harm. On the other hand, they have economic implications since PI-based regimens are substantially more expensive. Another unexpected result was that there were as many changes to PI-based regimens for toxicity as for switching criteria. These treatment changes to more expensive regimens should also be considered at the programmatic level regardless of the monitoring strategy.

This study had several limitations. First, although all patients satisfied contemporary WHO criteria for starting ARV therapy, none had a CD4 cell count below 50 cells/mm^3^ by protocol design, and fewer than anticipated primary endpoints were reached, many of them in the first 3 mo of follow-up before the switching strategy could take effect. When we compared outcomes occurring only after 3 mo, similar results were found. Even though our results are consistent with the observations made in the HBAC study and indicate that, at least over 3–5 y, monitoring by CD4 or VL leads to essentially the same outcome, they must be interpreted with caution. The long term outcomes, including response to second-line treatment have not been thoroughly studied. While the FDO score did not differ between arms, six out of 16 patients in the CD4-monitoring arm developed ≥3 NRTI mutations, in contrast to two out of 18 participants in the VL-monitoring arm. From this finding, had the study continued longer, less optimal response to second-line treatment may be observed when monitoring with CD4 only. The fairly similar resistance patterns at failure may be related, at least in part, to the low barrier to mutation toward high-level resistance in NNRTIs and 3TC. The same NNRTI and 3TC resistance mutations were probably selected in both arms, with only thymidine analogue mutation (TAM) accumulation differing, perhaps because of longer duration of failure in the CD4 arm.

The generalizability of our findings to routine care settings must also be considered: participants in this trial were seen and counseled every month throughout the entire study. The overall rates of virologic failure and loss to follow-up were lower than those reported in other settings, most likely due to close follow up. It is also important to note that laboratory evaluations were performed every 3 mo rather than every 6 mo as recommended by WHO. It is not clear how less frequent monitoring would have affected the outcomes in this study.

Economic evaluations, mostly in sub-Saharan Africa, have generated conflicting results regarding the cost-effectiveness of VL and CD4 monitoring strategies [29]. The DART analysis concluded that no form of laboratory monitoring was cost-effective in Uganda and Zimbabwe [30], while the HBAC analysis considered CD4 monitoring “desirable clinically and economically” [31]. Both rejected VL monitoring as not cost-effective. However, published analyses do not take fully into account the wider benefit of VL monitoring in supporting adherence and thus preventing drug-resistance or in reducing HIV transmission [29]–[35]. A preliminary report by Keiser et al. indicated a substantial improvement in cost-effectiveness of VL when the effect on adherence and HIV transmission were considered [36]. Moreover, it is possible that newly developed, point of care VL tests would further reduce the cost and increase the feasibility of routine VL monitoring in many settings [37].

In summary, at 3 y, rates of clinical failure and loss of treatment options did not differ between the two monitoring strategies, although the longer-term consequences of CD4 monitoring are unknown. These findings confirm that access to life-saving ARV treatment should continue to be expanded even in settings without virological monitoring, and provide reassurance to treatment programs currently based on CD4 monitoring alone, as VL measurement becomes more affordable and feasible in resource-limited settings.

## Supporting Information

Text S1 **List of hospital sites with number of patients enrolled.**
(PDF)

Text S2 PHPT-3 protocol.pdf.
(PDF)

Text S3 **CONSORT statement.**
(PDF)

## Abbreviations

ARV: antiretroviral
CDC: US Centers for Disease Control and Prevention
DSMB: Data and Safety Monitoring Board
FDO: future drug option
HAART: highly active antiretroviral therapy
IQR: interquartile range
NNRTI: non-nucleotide reverse transcriptase inhibitor
PHPT-3: Programs for HIV Prevention and Treatment
PI: protease inhibitor
ULN: upper limit of normal
VL: viral load
