# Supplementary material for: Switching HIV Treatment in Adults Based on CD4 Count Versus Viral Load Monitoring: A Randomized, Non-Inferiority Trial in Thailand
Source: PLoS Med. 2013 Aug 6;10(8):e1001494. doi: 10.1371/journal.pmed.1001494 (PMC3735458; doi:10.1371/journal.pmed.1001494)
Supplement: Text S1 — List of hospital sites with number of patients enrolled. (PDF) [file pmed.1001494.s001.pdf]

PHPT Hospitals sites and numbers of participants enrolled

Prapokklao Hospital (75), Chantaburi; **Rayong Hospital** (61), Rayong; **Mae Chan Hospital** (56), Chiang Rai; **Phayao Provincial Hospital** (53), Phayao; **Hat Yai Hospital** (50), Songkla; **Lamphun Hospital** (50), Lamphun; **Sanpatong Hospital** (48), Chiang Mai; **Samutsakhon Hospital** (45), Samutsakhon; **Chiangrai Prachanukroh Hospital** (34), Chiang Rai; **Chonburi Hospital** (32) Chonburi; **Maharat Nakonratchasima Hospital** (30), Nakhonratchasima; **Nakornping Hospital** (28), Chiang Mai; **Buddhachinaraj Hospital** (26), Pitsanuloke; **Ratchaburi Hospital** (25), Ratchaburi; **Lampang Hospital** (25), Lampang; **Mahasarakam Hospital** (23), Mahasarakam; **Bhuddasothorn Hospital** (15), Chachoengsao; **Samutprakarn Hospital** (11), Samutprakarn; **Chiang Kham** Hospital (11), Phayao; **Nong Khai Hospital** (10), Nong Khai; **Regional Health Promotion Centre 6-Khon Kaen** (8), Khon Kaen.
