# Supplementary material for: Switching HIV Treatment in Adults Based on CD4 Count Versus Viral Load Monitoring: A Randomized, Non-Inferiority Trial in Thailand
Source: PLoS Med. 2013 Aug 6;10(8):e1001494. doi: 10.1371/journal.pmed.1001494 (PMC3735458; doi:10.1371/journal.pmed.1001494)
Supplement: Text S2 — PHPT-3 protocol.pdf. (PDF) [file pmed.1001494.s002.pdf]

Research Protocol

Monitoring HAART in HIV-infected patients in Thailand**: Comparison of two strategies to monitor ARV treatment, based on CD4 cell count or viral load, to ensure optimal immunological and clinical outcome and preserve future treatment options in AIDS patients**

Summary

Implementation of highly active antiretroviral therapy (HAART) has led to a substantial decrease in HIV-related mortality and morbidity. With this important advance, however, has come a monitoring and decision-making process whose complexity challenges the medical care system, particularly in regions where there are large numbers of HIV-infected patients and relatively limited financial and health care resources. Current guidelines emphasize maximal and durable viral load (VL) suppression. However, while successful therapy is demonstrated by restoration of immunity, treatment failure is usually defined as the inability to maintain undetectable viral load, without regard to immune function. This situation often leads to a rapid sequence of therapeutic switches, thus narrowing therapeutic options over time. A monitoring strategy driven primarily by the patient's immune restoration would most likely be as effective in preventing disease progression, would lead to fewer changes in HAART regimens and would be considerably simpler and cost effective.

The primary objective is to compare the clinical outcomes of the standard antiretroviral monitoring strategy based on VL (VL-S), with a simpler strategy based on CD4+ cell count (CD4-S). An important secondary objective is to compare the ability of these two strategies to preserve treatment options.

The proposed study is a multicenter, Phase III, randomized, non-inferiority trial comparing VL-S with CD4-S among antiretroviral naive immunocompromised adults treated with a NNRTI-containing regimen in Thailand. The study will take place at 29 study sites and the study population will be 700 HIV infected women or men. Index cases will be women screened within the mother-child prevention program, and/or their partners with a CD4+ T cell count between **50** and 250/mm3. For women, randomization will take place after delivery. The initial HAART regimen will be a NNRTI containing regimen, the subsequent one will be a PI containing regimen. Under VL-S, switching is considered when VL rises above **400** copies/ml; Under CD4-S, switching is considered when a relative decline in the CD4+ cell count more than 30% from peak values **within 200 cells from baseline** is observed.

The primary endpoint for monitoring strategy comparison is clinical failure, defined as confirmed CD4 count below 50/mm^3, first or new AIDS-defining event, or death. A secondary endpoint is the number of drugs exhausted, taking into account cross-resistance mutations and shared toxicities. A pilot pharmacokinetic sub study will evaluate drug levels in the study population.

Principal Investigator

Dr. Marc Lallemant
IRD054/Perinatal HIV Prevention Trial
29/7-8 Samlan Road, Soi 1
Prasing, Muang, Chiang Mai
Tel: 0 53 814 633-8
Fax: 0 53 814 269
Email: marc@phpt.org

Dr. Sombat Thanprasertsuk
Bereau of AIDS, TB and STIs
Department of Disease Control
Ministry of Public Health
Tivanont Road, Muang
Nonthaburi 11000
Tel: 0 2590 3201
Fax: 0 25918413

Prof.Dr. Suporn Koetsawang
Reproductive Health Association of Thailand
41/9 Tarinee Pinklao Village
Baromrachachonnanee Road
Talingchan, Bangkok 10170
Tel: 0 2880 7084
Fax: 0 2880 7083

Co-investigators

Dr. Aram Limtrakul
Health Promotion Center Region 10, Chiang Mai
51 Prachasumphan Rd., Muang
Chiang Mai 50100
Tel: 0 5327 6856
Fax: 0 5327 4014

Dr. Nuananong Wirayutwatthana
Lamphun Hospital
Muang
Lamphun 51000
Tel: 0 5351 0020-23
Fax: 0 5351 0023

Dr. Guttiga Halue
Phayao Provincial Hospital
Muang
Phayao 56000
Tel: 0 5443 1209
Fax: 0 5448 1708

Dr. Pacharee Kantipong
Chiangrai Prachanukroh Hospital
Muang
Chiang Rai 57000
Tel: 0 5371 1300
Fax: 0 5371 3044

Dr. Yuwadee Buranawanitchakorn
Chiang Kham Hospital
244 M. 4 Tambon Yuan, Chiang Kham
Phayao 56110
Tel: 0 5445 1300-1
Fax: 0 5441 6615

Dr. Vanichaya Luedee
Phan Hospital
Phan
Chiang Rai 57120
Tel: 0 5372 1345
Fax: 0 5372 1346

Dr. Chanthana Jongpipan
Mae Sai Hospital
101 M. 10 Tambon Wiangbangkham, Mae Sai
Chiang Rai 57130
Tel: 0 5373 1301
Fax: 0 5364 2510 ex.2345, 731 300-1 ex.2345

Dr. Praparb Yuthavisuthi
Prapokklao Hospital
38 Leibnern Rd., Muang
Chantaburi 22000
Tel: 0 3932 4975
Fax: 0 3931 1511

Asst. Prof. Chureeratana Bowonwatanuwong
Chonburi Hospital
69 M. 2 Sukumvith Rd., T.Ban Suan, Muang
Chonburi 20000
Tel: 0 3827 4200 -7
Fax: 0 3827 4911

Dr. Surabhon Ariyadej
Rayong Hospital
Muang
Rayong 21000
Tel: 0 3861 1104
Fax: 0 3861 2003

Dr. Banyat Chetanachan
Klaeng Hospital
254 Sukumvit Rd., Klaeng
Rayong 21110
Tel: 0 3867 1113-4
Fax: 0 3867 1113-4 ex. 530

Dr. Pakorn Wittayapraparat
Chacheongsao Hospital
Muang
Chacheongsao 24000
Tel: 0 38 511 033
Fax: 0 3851 1633

Dr. Prattana Leenasirimakul
Nakornping Hospital
Mae Rim
Chiang Mai 50180
Tel: 0 5389 0755-64
Fax: 0 5322 2152

Dr. Sinart Prommas
Bhumibol Adulyadej Hospital
Phaholyothin Rd., Saimai,
Bangkok 10220
Tel: 0 2 534 7000
Fax: 0 2 523 8873

Dr. Vichien Attakornwatana
Somdej Pranangchao Sirikit Hospital
Plutalaung, Amphur Sattaheep
Chonburi 20180
Tel: 0 3824 5735-65
Fax: 0 3824 5771

Dr. Somboon Tunsuphasawasdikul
Buddhachinaraj Hospital
91 Srithamapidok Rd., Muang
Pitsanuloke 65000
Tel: 0 5521 9844
Fax: 0 5525 8813

Dr. Narong Winiyakul
Regional Health Promotion Centre 6, Khon Kaen
195 Srichan Rd, Naimuang, Muang
Khon Kaen 40000
Tel: 0 4323 6772
Fax: 0 4324 3416

Dr. Apichat Chutanunta
Samutsakorn Hospital
1500 Akekachai Rd., T. Mahachai, Muang
Samutsakorn 74000
Tel: 0 3442 7099-104
Fax: 0 3441 1488

Dr. Naree Eiamsirikit
Samutprakarn Hospital
71 Chakrapak Rd., T. Nai Muang
Samutprakarn 10280
Tel: 0 2701 8132-9
Fax: 0 2389 5921

Dr. Suchat Thongpaen
Mahasarakam Hospital
1105 Padungwithee Rd., T. Talad Muang
Mahasarakam 44000
Tel: 0 4374 0993
Fax: 0 4371 1433

Dr. Wichai Panitsuk
Ratchaburi Hospital
Muang
Ratchaburi 70000
Tel: +66 (0) 32 327 901
Fax: +66 (0) 32 321 825

Dr. Rittha Lertkoonlak
Maharaj Nakornratchasima Hospital
49 Changpuag Rd, Muang
Nakornratchasima 30000
Tel: 0 4425 4990-2
Fax: 0 4424 6389

Dr. Surachai Pipatnakulchai
Pranangklao Hospital
206 Nonthaburi 1 Rd., Bangkrasaw, Muang
Nonthaburi 11000
Tel: 0 2527 0246 ext. 8410,01
Fax: 0 2 526 5629

Dr. Ampaipith Nilmanat
Hat Yai Hospital
Amphur Hat Yai
Songkla 90110
Tel: 0 7423 0800-4
Fax: 0 7424 6600

Dr. Naruphon Yuthakasaemsan
Nong Khai Hospital
Meechai Rd, Naimuang, Muang
Nong Kai 43000
Tel: 0 4246 1088
Fax: 0 4242 1465

Dr. Pramual Thaingamsilp
Kalasin Hospital
283 Kalasin Rd., T. Kalasin, Muang
Kalasin 46000
Tel: 0 4381 1020
Fax: 0 4381 2962

Dr. Ruangyot Thongdej
Kranuan Crown Prince Hospital
1 M.11 Suebsarakam Rd., Nonggo, Kranuan
KhonKaen 40170
Tel: 0 4325 1661
Fax: 0 4325 1302

Dr. Boonyong Jeerasuwannakul
Roi-et Hospital
111 Ronnachai Chanyuth Rd., T. Nai Muang,
Muang, Roi-et 45000
Tel: 0 4351 1336
Fax: 0 4351 1503

Dr. Panita Pathipvanich
Lamphang Hospital
Muang
Lamphang 52000
Tel: 0 5422 3623-7
Fax: 0 5422 6126

Research Plan

A. SPECIFIC AIMS

Current management of antiretroviral (ARV) therapy emphasizes stringent viral load monitoring to ensure maximal and durable viral load suppression [13-16]. This strategy is costly and labor intensive, and often leads to a rapid sequence of therapeutic switches with the consequence of narrowing therapeutic options over time[17].

The proposed study is based on the hypothesis that a monitoring strategy driven primarily by the patient's immune restoration would be equally effective in preventing disease progression, would be simpler to implement, and would lead to fewer antiretroviral regimen changes thereby potentially saving treatment options for patients over time as well as reducing the cost and complexity of care.

This study aims to determine whether it is necessary to measure and follow viral loads to achieve optimal results of HAART in a population of therapy-naïve patients, or if it is possible, or even preferable, to use only the CD4 cell count as a guide in therapeutic decision-making. The proposed comparison of simplified vs. standard monitoring schemes may be analogous to the comparison of shorter and longer zidovudine use to prevent mother-to-infant HIV transmission in Thailand, conducted by our group in 1997-99 [N Engl J Med 2000, 343:982-991]. The results of that study have helped to shape the public health approach to mother-to-infant HIV transmission prevention in Thailand, and it is hoped that the results of this proposed study will have a similar effect on treatment of HIV-infected adults.

The proposed study is a multicenter, phase III, randomized, controlled, non-inferiority trial comparing a simplified CD4 based antiretroviral monitoring strategy with the standard viral load based monitoring strategy among antiretroviral naive immunocompromised adults treated with a NNRTI followed by a PI-containing regimen in Thailand. The study will enroll 700 HIV infected women or men. The women will be screened during pregnancy and enrolled in the study in the postpartum period. Their partners, if consenting, will be screened at the same time as the women, or as soon as possible thereafter and enrolled in the study as soon as they are found to qualify. Eligible subjects will have a CD4+T cell count (further denoted CD4) between **50** and 250/mm^3.

The study will be carried out in 29 hospitals throughout Thailand (mainly in Bangkok and the eastern and northern provinces of Thailand) as a collaborative effort between Harvard and Columbia Universities, the Ministry of Public Health of the Kingdom of Thailand, Mahidol, Chiang Mai and Khon Kaen Universities, and the Institut de Recherche pour le Développement (IRD) in France. These sites are currently participating in the study conducted by the "Perinatal HIV Prevention Trial group" (referred to throughout this proposal as PHPT), a trial of nevirapine (NVP) plus zidovudine (ZDV) for the prevention of mother-to-infant transmission of HIV (R01 HD 39615).

Primary objective:

To compare the clinical outcomes of a monitoring strategy based on CD4 count (further denoted CD4-S) with the standard monitoring strategy based on viral load (VL-S), among antiretroviral treatment naive immunocompromised adults initiating therapy with a NNRTI-containing regimen.

Secondary objectives:

To compare the ability of the two monitoring strategies to spare ARV treatment options, taking into account the number of therapeutic options left and the resistance profile.

To evaluate the safety and tolerance of highly active antiretroviral therapies in immunocompromised Thai adults.

To analyze the relative contribution of lack of adherence, resistance patterns, drug exposure, intolerance, and toxicities, to treatment modifications, and virological and immunological failure.

B. BACKGROUND AND SIGNIFICANCE

B.1 Management of combination antiretroviral regimens for the treatment of HIV/AIDS

Since the mid 1990s, use of combination antiretroviral treatment regimens, also referred to as Highly Active Antiretroviral Therapy (HAART), has radically modified AIDS prognosis [18, 19]. These combinations are capable of decreasing viral load to extremely low levels and have been associated with dramatic decreases in morbidity and mortality. As the eradication of HIV infection cannot be achieved with currently available antiretroviral regimens, the objective of therapy is maximal and durable suppression of viral replication [20]. Such suppression may then achieve the goals of restoration and/or preservation of immunological function, improvement of quality of life, and reduction of HIV-related morbidity and mortality [19, 21-25]. However, as the concepts of HIV disease management continue to evolve and develop with the advent of new studies and their findings, treatment guidelines are continuously updated. The following is a brief summary of the current consensus as reflected in the 2001 update of the Department of Health and Human Services (DHHS) guidelines regarding treatment objectives, criteria for initiation of therapy, and monitoring and change of therapy strategies [14].

Initiation of therapy: Treatment is generally offered to patients who are symptomatic or who have fewer than 350 CD4+ cells/mm^3 or plasma HIV RNA levels exceeding 30,000 copies/mL (bDNA assay) or 55,000 copies/mL (RT-PCR assay), based on the willingness and readiness of the individual to begin therapy; the potential benefits and risks of initiating therapy in asymptomatic individuals; and the likelihood, after counseling and education, of adherence to the prescribed treatment regimen.

For asymptomatic patients with CD4+ cell counts >350 cells/mm^3, the consensus is less clear. Robust immune reconstitution occurs in most patients who initiate therapy in this CD4 count range and viral suppression may be easier to achieve and maintain. However, toxicities [26-31] and adherence challenges may outweigh benefits of therapy [32, 33]. Some studies have found sex-specific differences in viral load and CD4 counts; however it seems that the rate of disease progression does not differ in a sex-dependent manner [34-36].

Monitoring: After therapy is initiated, decisions regarding changes are guided by monitoring viral load and CD4 cell counts as well as evaluating the clinical condition of the patient. Although individual responses are variable, antiretroviral therapy leads to increases in the CD4 count of 100-200 cells/mm^3 or more, a response generally related to the degree of viral load suppression [19, 21, 24, 25, 37-41]. Partial reconstitution of immune function induced by HAART may allow for discontinuation of prophylaxis for some opportunistic infections [42-44]. A favorable CD4 cell response can occur with incomplete viral load suppression [12, 45-47]; conversely, probably because of intercurrent opportunistic infections or perhaps as a form of drug toxicity, the CD4 count can remain stable or decrease even with complete viral suppression [47, 48].

Although 70-90% of antiretroviral drug-naive patients achieve viral load suppression 6-12 months after initiation of therapy, only 50% of ARV experienced patients achieve similar results [25, 41, 49, 50]. The rate of viral load suppression is associated with the baseline CD4 cell count, the initial viral load, the potency of the regimen, adherence to the regimen and drug pharmacokinetics, prior exposure to antiretroviral agents, and the presence of opportunistic infections. Adherence is one of the most important determinants of the degree and duration of virological suppression [32, 51, 52]. In treatment naive patients, non-adherence is usually the cause of the first treatment failure. A high degree of adherence is necessary for optimal virological suppression with HAART; several studies have shown that 90-95% of doses must be taken for optimal suppression [32, 53]. Therefore, when proceeding with treatment decisions for patients who are failing therapy, adherence should first be assessed to clarify whether the solution is to switch therapies or to reinforce and improve adherence.

Drug resistance: Complexities in managing HAART are compounded by failures in suppressing HIV replication in treatment-experienced patients, often an indication of drug resistance. When virological failure has been shown to be associated with resistance to only one component of the regimen, it may be possible to substitute individual drugs in a failing regimen [54]. However,

resistance to more than one agent often emerges during therapy. Of further concern is the possibility of broad cross-resistance among drugs within the same class [55-57].

Drug resistance is detected through genotyping or phenotyping assays[1-9]. Genotyping assays detect drug resistance mutations that are present in the relevant viral genes (i.e. reverse transcriptase and protease). Their interpretation requires knowledge of the various antiretroviral drug related mutations and their potential for cross-resistance [see Section C.6. for Assessment of resistance].

Phenotypic drug susceptibility assays have the advantage that they directly measure the susceptibility of a patient's virus to antiretroviral drugs. Three commercial tests are available today (ViroLogic's Phenosense-HIV, Virco's Antivirogram, and Viralliance Phenoscript), all of which use the technology of HIV-1 resistance test vectors. The vectors are constructed by insertion of amplified patient-derived RT and PR viral gene segments into a modified HIV-1 genome that carries an indicator gene. Virus particles, produced by transfecting host cells, are used to infect target cells (laboratory cell lines such as MT4 or 293). Drug susceptibility is measured by comparing indicator gene activity in the presence of varying concentrations of antiretroviral drugs. Phenotypic testing is slower and considerably more expensive. Neither phenotypic nor genotypic testing detects minor populations of drug-resistant virus.

Phenotypic testing has not been validated with the CRF01_AE subtype in Thailand. Moreover, despite the intrinsic attractiveness of phenotypic testing, the Narval Trial (ANRS 088), which assessed the respective value of phenotypic versus genotypic testing versus clinical judgment for guiding drug switching and antiretroviral choice, showed that both tests performed about the same, with only a slight benefit of the genotyping test over standard of care and of the phenotyping test in patients with limited protease inhibitor experience [58].

Several groups have examined antiretroviral resistance and resistance testing in non B HIV-1 subtypes including subtype E (CRF01_AE) [4, 5, 8, 59-63]. Clinical isolates from drug-naïve patients display a high natural polymorphism of the RT and protease genes which does not appear to affect the susceptibility of those viruses to ARV compared to B-subtypes [64, 65]. No major new mutations linked to NRTI or protease resistance have been found, although some accessory drug resistance mutations have been described in non-B subtypes which are distinct from those in B subtypes. The clinical significance of this is not yet completely known [59].

In ARV experienced patients, Sato and al. and Phanuphak have shown that changes in amino acids coded by the RT gene from strains recovered after treatment with ZDV and/or ddI were identical for isolates of E subtypes to those reported for B subtypes [63]. Even though the pathway to resistance may vary, so far mutations associated with resistance to NRTI, NNRTI or PI appears to be very similar in isolates of B- and E-subtypes.

Viral "Fitness": In the presence of incomplete viral suppression, antiretroviral drug pressure selects not only for HIV resistance but, at least in some cases, the emergence of viral strains with reduced replicative capacity. This replicative impairment may be reversed, and fitness may improve as more mutations accumulate [66]. Viral virulence, loosely defined as the ability to deplete CD4+ cells in vivo or in vitro, may also be affected by genomic changes in *gag/pol*. How drug resistance, viral fitness, and viral virulence combine to affect CD4+ cell depletion is not well understood. Understanding these relationships and the factors that influence them may allow clinicians to better manage antiretroviral therapy.

Assays to measure replicative capacity are under development and include competitive growth cultures with replication competent virus [67-70] and single replication cycle recombinant virus assays. Assays that attempt to measure virulence/pathogenicity are also under development and include in vivo animal models (SCIDhu mouse) [71] and ex vivo, three-dimensional, human lymphoid histoculture systems [72]. These assays have not been studied in prospective (or any other) clinical trials.

Switching antiretroviral therapy: Criteria for considering therapy changes include: insufficient reduction in plasma HIV RNA by 4-8 weeks following initiation of therapy; failure to reach undetectable levels of HIV RNA in plasma within 4–6 months of initiating therapy, repeated

detection of virus in plasma after initial suppression to undetectable levels (suggesting the development of resistance); confirmed increase of plasma HIV RNA not attributable to concurrent infection or vaccination; persistently declining CD4 cell numbers, as measured on at least two separate occasions; intolerance/toxicity; or clinical deterioration. Other considerations include: adherence to medications; remaining treatment options; and preparation of the patient for the implications of the new regimen that includes side effects, drug interactions, dietary requirements and the possible need to alter concomitant medications. If viral suppression has been achieved but the patient experiences intolerance or toxicity, the drug involved should be changed, preferably replaced by a drug of the same class with a different tolerance or toxicity profile.

Therapies: In the mid 1980s placebo-controlled trials of ZDV established that antiretroviral treatment provided clinical benefit to HIV-infected individuals with advanced HIV disease [73]. Later, combinations of 2 Nucleoside Reverse Transcriptase Inhibitors (NRTI) provided additional, more durable clinical benefit [74]. The addition of Protease Inhibitors (PI) or Non Nucleoside Reverse Transcriptase Inhibitors (NNRTI) demonstrated further benefit [75-77]. Most guidelines recommend a combination of 3 ARV drugs [14, 78]: two NRTIs with one PI, one NNRTI or another NRTI. Appendices 6 and 9 provide a detailed outline of the current ARV combinations approved by the FDA with additional information relevant to toxicities, drug associations and interactions, pharmacokinetics and dosing schedules [see also sections C.2.1 Selection and Enrollment of Subject,]

Host genetics, disease progression and response to therapy: Several studies have shown an association between disease progression and host genetics, in particular genetic polymorphism involved in the immune response. For example, certain MHC or MBL gene alleles have been associated with the severity of infectious diseases such as Hepatitis B, C and HIV [79-84]. More recently, chemokines receptors/HIV co-receptor gene polymorphism [85-87] has been studied in relation to HIV disease susceptibility and progression, the CCR5 delta 32 deletion for example. This deletion results in the production of a protein that is non functional as chemokine as well as HIV receptor. While homozygous individuals for this mutated gene are highly, but not totally, protected against HIV infection, HIV infected heterozygous individuals progress more slowly to AIDS [88]. Natural variants of the CCR5 promotor gene have also been associated with modifications of HIV disease progression [89-92]. Natural ligands of the CCR5 genes (Mip 1a, Mip 1b and RANTES) as well as some alleles of the RANTES gene may also be associated with susceptibility or disease progression [93, 94]. Associations with other receptors or cytokine genes are also being studied [95-98]. Pharmacogenetics is an also rapidly expanding research field and recently, important physiological interactions involving different ARV drugs, have been reported. A report from the Swiss HIV Cohort study demonstrated a significant difference in drug concentrations of ARV drugs between patients with different allelic variants of the Multidrug-resistance transporter gene MDR1 [99]. The frequency of this functional polymorphism is significantly influenced by ethnicity; however, Caucasian and Asian populations have a similar polymorphism distribution [100]. The NNRTIs and PIs are metabolised by the hepatic cytochrome P450 enzyme system, which is composed of several subfamilies with several different isoforms. CYP3A4 is one of the predominant P-450 isoforms. Several allelic variants of CYP3A4 have been identified but no clear relationship between variants and drug disposition has been observed [101]. Also, abacavir hypersensitivity has been related to genetic factors in a Caucasian population [102], but their predictive values across populations are unknown.

C. RESEARCH DESIGN AND METHODS

C.1 Rationale

According to our current understanding of HIV disease pathogenesis, the optimal way to delay progression to disease is to completely suppress viral replication. Theoretically, any regimen that does not fully suppress viral replication (currently defined as a viral burden below 50 copies/mL on standard assays) will allow for the selection of resistance mutations [119, 120] leading to resistance to the current drugs and ultimately cross-resistance to other drugs in the same class.

However the objective of total viral suppression is not achieved in all individuals who receive HAART. In clinic populations, the rate of virological failure after 24-48 weeks of first-line HAART varies from 20 to 40%. In clinical trials, failure rates may be lower, in the range of 15-20% [19, 121]. Moreover, patients with virological failure in the initial regimen have lower rates of complete viral suppression with subsequent regimens [41, 49, 122, 123.]

Virological failure is not necessarily associated with immunological or clinical failure. For example, it has been noted that in patients who experience virological rebound while taking a PI-based regimen, viral load rarely returns to pre-treatment levels [124]. This indicates that, at least for a period of time, under continuing drug pressure, resistant viral strains less able to replicate persist, as has been demonstrated in vitro [125, 126].

The proposed protocol is designed to determine whether it is necessary to measure and follow viral loads to achieve optimal results of HAART in a population of therapy-naïve patients, or if it is possible, or even preferable, to use only CD4 counts as a guide to therapeutic decision-making. A strategy basing treatment decisions on viral load measurements and attempting complete viral suppression in all patients may lead to fast and multiple changes in therapy. In contrast, a monitoring strategy based on the evolution of the CD4 count allowing for a certain level of replication before changing therapy may be safe and more effective in preserving future treatment options, provided that the immune system is not harmed.

Two direct observations support the practice of monitoring antiretroviral therapy based on CD4 count:

1) For patients taking HAART, current CD4+ cell count is a better measure of short-term risk of disease progression than viral load [127-130]. 2) The sustained CD4 T-cell benefit in patients with rebounding viremia during PI therapy appears to predict a sustained clinical benefit [12,124, 131-133].

The Swiss HIV Cohort Study, for example, examined the positive effect of HAART even in the absence of optimal control of viremia. Patients were categorized based on their virological response to therapy: virological responders without rebound, patients with an initially undetectable viral load followed by a viral rebound, and patients who never reached an undetectable viral load. Clinical progression in all three groups was rare over 2 years of follow-up, even in patients with evidence of ongoing viral replication [124].

The virological mechanism underlying the preserved virologic and immunologic benefit despite the emergence of drug resistant virus remains unclear and is likely to be multifactorial [134]. Several virus- and host-related factors may be involved including pharmacokinetics, adherence, and tissue penetration; viral replicative capacity or “fitness”; generation and maintenance of an effective HIV-specific immune response; reduced generalized immune activation and, as a consequence, reduced availability of susceptible target cells; and maintenance of gains in immune reconstitution despite the persistence of detectable viremia.

To illustrate our genuine uncertainty about the outcomes of the proposed monitoring strategies, we simulated a scenario where a population initiating HAART would be followed for three years while submitted to either VL-S where the switching point is reached when VL rises above **400** copies per mL, or to CD4-S, where the switching point is reached when CD4 count declines more than 30% from peak values within **200 cells from baseline**. This simplified scenario assumes that under VL-S, the time under HAART spent while in virological failure is short, that new regimens are invariably successful, at least initially, and that there are no drug/family overlap between first and subsequent regimens.

Based on published data [19, 39, 41, 50, 121, 124, 129, 130, 135], we assumed a rate of virological failure of 25% yearly on the initial HAART regimen and a 35% yearly rate on any subsequent regimen. We also assumed that the rate of immunological failure would be 7%, a figure consistent with our observation in the cohort of patients receiving NNRTI based HAART in the PHPT hospital networks, and recent data would suggest an extended immunological benefit under a PI based treatment regimen even in the presence of viral replication [49, 136-138]

With the above assumptions, at the end of a three year follow-up, approximately 42% of the patients randomized in the VL-S monitoring arm would still be on the initial therapy, 37% on the second line regimen, 18% on the third and 3% on the fourth. The average number of switches would have been 0.8 per patient. Finally, it would be expected that the percentage of time spent on each HAART regimen would have been 65% for the initial regimen, 26% for the second line, 7% for the third and 1% for the fourth.

The results under the CD4 monitoring strategy would be strikingly different: approximately 88% of the patients would still be under the initial regimen after 3 years, and 11% under the second line regimen. The average number of switches would have been 0.2 per patient, 88% of the time on HAART would have been spent under the initial regimen and 12% under the second line regimen.

This scenario would suggest that patients monitored based on CD4 count are at advantage compared to patients monitored based on viral load. However, a main concern about delayed switches is the sequential accumulation of drug resistance mutations that may diminish the chances of viral re-suppression with successive HAART regimens. As described in the background section, the emergence of drug resistance mutations after clinical detection of viral rebound is variable. In general, the isolated lamivudine (3TC) and non-nucleoside reverse transcriptase inhibitor (NNRTI) mutations occur soon after viral rebound, whereas mutations associated with resistance to nucleoside analogues (except 3TC) and protease inhibitors (PIs) emerge after more prolonged periods of virologic relapse [54, 139, 140]. Although it may be hypothesized that subjects remaining on a partially suppressive regimen will accrue additional resistance mutations to the current regimen, it is also likely that a substantial proportion of patients switched early will experience virologic rebound on their new regimen. The relative risk of the accumulation of more mutations to an “old” regimen versus the development of resistance to “new” drugs requires evaluation in a randomized trial. Moreover, understanding why patients continue to maintain some degree of partial viral suppression despite the emergence of drug resistance will allow for the development of therapeutic strategies aimed at determining when to switch, when to continue, and when to stop antiretroviral therapy.

The evaluation of a simpler monitoring strategy is particularly relevant in Thailand. With recent global emphasis on the treatment of infected individuals, the large number of candidates for such programs will place an equally large burden on the medical system. Of the various components of this burden, cost of drugs, which has decreased dramatically over the past two years, is only one part of the problem. Of at least equal, and perhaps greater, importance is the education of health care practitioners and patients in the complex management of multiple-drug HIV therapy, equipping of laboratories, training of laboratory personnel, and finally implementation and cost of a system for monitoring viral load and CD4 count. As the country is embarking on this ambitious program, it is of utmost importance to determine the ARV management strategy that will be most beneficial in the long term for patients in the Thai context [See section C.15. ARV management strategy for implementation in the Thai Context].

Assessing simplified yet effective monitoring strategies will also be important for countries less advanced than Thailand. The year 2001 marked a turning point in the approach to AIDS prevention and care with the increased availability of generic drugs, the agreement by five large pharmaceutical companies to significantly reduce the cost of antiretroviral drugs, and a pledge by the international community at the 26th special session of the United Nations General Assembly to drastically increase its financial and technical support to poor countries affected by AIDS (UN, June 2001). Universal access to antiretroviral therapies will be one of the major public health undertakings of the next decade; yet few physicians are skilled in the use of antiretrovirals. In many situations, the health infrastructure is not fully prepared to train health personnel, distribute the drugs, inform and evaluate patients, initiate therapy and monitor viral response, immune status or side-effects of the drugs [141]. Current guidelines from the World Health Organization emphasize the role of clinical and immunological monitoring of antiretroviral therapy [142]. Groups working in developing countries are assessing strategies to optimize resources and increase patients’ chances for a sustainable response to antiretroviral therapy [143, 144]. In this context, the

documentation, by comparison to current standards, of a CD4 based monitoring strategy that may be equally safe, more practical, and spare more future treatment options will be extremely useful.

In summary: If patients are immunologically well and can tolerate their current therapy, avoiding premature switches is likely to expose them to fewer regimens, thus averting new toxicities, new drug interactions, and new adherence issues. It also has the potential of preserving more drug options for current as well as future use. Thus, besides the obvious benefit of demonstrating the effectiveness of a monitoring strategy that is likely to be easier to implement in resource limited regions of the world most affected by HIV, we have a genuine uncertainty as to which strategy would be more beneficial in terms of clinical outcome and therapeutic options spared. This question can only be resolved through a carefully monitored clinical trial.

C.2. DESIGN

The proposed study is a multicenter, Phase III, randomized, non-inferiority trial comparing the standard viral load based antiretroviral monitoring strategy with a simpler CD4 based monitoring strategy among antiretroviral naive immunocompromised adults treated with a NNRTI-containing regimen in Thailand. In 29 study sites, the study population will be 700 HIV infected subjects (women screened during pregnancy, and/or their partners) who have a CD4 count between **50** and 250/mm^3.

Within each study site and patient stratum (postpartum women and male partners), subjects will be randomly assigned to one of two monitoring strategies:

- VL-S, the standard viral load (VL) based monitoring strategy, where switching is performed when VL is confirmed (within one month) above **400** copies per mL.
- CD4-S, the alternative CD4 based monitoring strategy where switching is performed when a confirmed (within one month) relative decline in CD4 count of more than 30% from peak values **is observed within 200 cells from baseline**.

The initial HAART regimen will be a NNRTI+NRTI containing regimen and the second line regimen will be a PI containing regimen, subsequent regimens will be chosen individually based on tolerance, previous drugs used, resistance profile, and drugs available. Patients will be followed until the end of the study (5 years for the first enrollee, three years for the last enrollee).

For patients who reach their predefined viral load or CD4 threshold for switching, laboratory results will be confirmed within one month and, before any change of therapy is made, causes for viral rebound or immunological deterioration will be investigated, with particular attention to adherence or tolerance issues, toxicities and co-infections. Upon confirmation of laboratory results, the treatment regimen may be switched to the next scheduled regimen.

C.2.1. Selection and Enrollment of Subjects

The study population is HIV-infected women identified through the MOPH Prevention of Mother to Child Transmission (PMTCT) program and/or their partners, consulting at one of the study sites, with a CD4 count between **50** and 250/mm^3 and who otherwise satisfy entry criteria. Based on current figures of HIV prevalence at the study sites and enrollment in PHPT-2, we estimate that about 1500 HIV pregnant women will be willing to undergo pre-enrollment evaluation. After appropriate counseling, pregnant women will be encouraged to invite their partner to come to the hospital for information, counseling, and HIV testing. Participation of both women and their partner in the study will be encouraged if they meet inclusion criteria, but joint participation will not be a prerequisite for enrollment of either one. Data from our previous trials suggest that about 21% of those women will meet entry criteria.

Pre-enrollment evaluations for the women will take place during pregnancy and as soon as possible for their partner. Standard ARV prophylaxis for the prevention of mother to child HIV transmission will be provided to all pregnant women. If the clinician judges that a woman needs to start HAART during pregnancy, after careful evaluation of the expected risks and benefits HAART will be offered, but she will not be randomized in the study. If nevirapine is used during labor for

PMTCT, theoretical considerations would warrant starting maternal HAART right after delivery in order to prevent emergence of NVP resistance mutations. Moreover, a previous study in Thailand has shown that women in PMTCT programs hope to continue ARV therapy for their own health after delivery [145]. Taking into account these considerations, women who qualify for study entry will be counseled during pregnancy, and if they appear ready and willing to start they will be encouraged to initiate therapy as soon as possible after they have delivered.

HIV infected women and partners who do not fulfill enrollment criteria will be followed on an outpatient basis to receive counseling and monitoring of their health condition. Patients with CD4 above 250 cells per mm^3 will have a CD4 count performed every six months. If the CD4 count drops below 250 cells per mm^3, enrollment in the study will be proposed to the patient. Patients with a CD4 count lower than **50** cells per mm^3 will receive treatment through the MOPH program but will not be eligible for randomization in the study.

Justification for implementing this study in the Mother and Child Health framework

Following the results of PACTG 076 [114, 146] and the clinical trials conducted in Thailand showing the efficacy of ZDV prophylaxis for the prevention of perinatal transmission of HIV, the Health Promotion/Mother and Child Health Division of the Department of Health and the Department of Communicable Diseases Control of the MOPH implemented a nationwide program to prevent perinatal HIV transmission, with universal access to counseling, HIV testing, ZDV prophylaxis and infant formula feeding. This program has already led to a decline in the number of reported pediatric AIDS cases. The proposed study builds upon this success, using the Mother and Child Health framework as an entry point for initiating therapy in immunocompromised HIV infected women and/or their partners. In addition, antenatal clinics are the only location where adults are systematically screened for HIV infection, and many women, and their partners, learn in this setting for the first time of their HIV status.

In April 2001, at the end of the Third International Symposium on Pediatric AIDS in Thailand organized by PHPT and the Ministry of Public Health, participants decided to submit a Declaration to the Minister of Health requesting antiretroviral treatments for immunocompromised women diagnosed during pregnancy. In November, 2001 the Ministry of Public Health officially designated Mother and Child Health as the first priority for its expanded access program to antiretrovirals [See Section D15. ARV management strategy for implementation in the Thai context]. Parents are a particularly appropriate target for therapy because of the importance of their role as parents of both infected and uninfected infants.

Justification for the chosen regimens

The chosen HAART regimen for initial therapy is a 2NRTIs+NNRTI containing regimen specific components of which, d4T or ZDV or TDF or 3TC/FTC, or NVP or EFV, will be selected depending on the patients needs (toxicity, sex, co-infections, tolerance) and availability. Such a NNRTI containing regimen appears most appropriate because: 1) The recently published AACTG 384 [N Engl J Med 2003;349:2293-303] has demonstrated that a NNRTI compared to a PI containing regimen gave optimal results as first line therapy; 2) NNRTI based regimens are now recommended as first line therapies by both the Thai and International treatment Guidelines.

The second line regimen will be a PI containing regimen: 2NRTIs selected based on the first line and indinavir boosted with ritonavir (IDV/r). Both regimen are appropriately potent combination in the **50**-250 CD4 range; [11,75, 76, 122, 147-149]; GPOvir (d4T/3TC/NVP), 3TC, NVP, EFV, ZDV and IDV/r are available in Thailand[10] with convenient bid dosing. Through reduced dosing because of patients' lower average weight for IDV/r, it is the most inexpensive PI based regimen in Thailand. Subsequent regimens will be chosen individually based on tolerance, previous drugs used, resistance profile, and drugs available.

C.2. 2. Eligibility, Inclusion and Exclusion Criteria

Eligibility Criteria. Patients will be eligible if they have confirmed HIV-infection, intend to be followed at a study site for the duration of the study, can provide informed consent, and, for women, are

willing not to breastfeed and to comply with an effective contraception method for the duration of the study.

Inclusion Criteria. Written informed consent (if age<18 years, legal guardian's consent and patient's assent); HIV-1 infection documented by two HIV antibody tests on two different blood draws; confirmed CD4+ cell count **above 50** and lower than 250 cells/mm^3 **within 6 months** prior to study entry; naive to antiretroviral therapy (except exposure to zidovudine during pregnancy and/or nevirapine during labor); willingness to initiate, modify, or stop antiretroviral therapy in accordance with the randomized monitoring scheme assignment; subject's understanding that study drugs will be supplied only during participation in the study, and reasonable certainty that he/she will be able to access HIV treatment after the study.

Exclusion Criteria. For women, pregnancy. Current active substance or alcohol abuse that would interfere with participation in the study. Chemotherapy for active malignancy. Active opportunistic infection and/or serious bacterial infection or unstable or severe medical condition within 14 days before randomization. Chronic malabsorption or chronic diarrhea (> 6 loose stools/day for > 14 days within one month of entry) or recent (within 7 days) unresolved acute diarrhea. The following laboratory values: hemoglobin < 8.0 mg/dl, absolute neutrophil count < 1000 cells/mm3, ALT, AST or total bilirubin value > 5.0 x ULN, serum creatinine > 1.0 x ULN, platelet count < 50,000/mm3, pancreatic amylase >2.0 x ULN, or total amylase > 2.0 X ULN plus symptoms of pancreatitis. Pre-existing diabetes mellitus (prior gestational diabetes is allowed). Acute hepatitis within 30 days of study entry. Any clinically significant diseases (other than HIV infection) or clinically significant findings during screening medical history or physical examination which, in the investigator's opinion, would interfere with the conduct of the study. Psychosocial environment or condition which, in the physician's opinion, makes adherence to the protocol highly unlikely.

HIV infected women and partners who are not willing to participate in the study or those who do not fulfill enrollment criteria, in particular those with a CD4+ cell count lower than **50**, will receive counseling on HIV/AIDS treatment and will be cared for by the MOPH Mother and Child Health services. In December 2001, the MOPH launched a new program to provide ARV treatment through the Mother and Child Health care delivery system to women diagnosed with HIV during pregnancy and their partners. Patients with CD4+ cell count above 250 will continue to have a visit and a CD4+ cell count every six months for the duration of the enrollment period. If the CD4+ cells count drops below 250/mm^3, enrollment in the study will be considered.

Allowed/Disallowed Medications.

The list of contraindicated associations allowed and disallowed medications for each antiretroviral drug is provided in the Appendix 9 (Toxicity grading and management) and Appendix 10 (Drug regimens).

Opportunistic Infections (OI) Prophylaxis: Prophylaxis and treatment of OIs (in particular PCP, tuberculosis, cryptococcal meningitis, and *P. marneffei*, the most frequent OIs in Thailand) will follow the Thai recommendations regularly adapted from international guidelines including recommendations on the management of tuberculosis treatment and HAART [150].

C.3 Study Visits and Laboratory Evaluations

The pre-enrollment visit for women will take place as early as possible after routine prenatal counseling and HIV testing for the prevention of Mother to Child transmission of HIV. Evaluations, enrollment and HAART initiation will take advantage of the routine follow-up schedule of visits for women participating in the national perinatal HIV prevention program. As much as possible, study visits after delivery will follow the routine schedule of postpartum care. Visits for the woman and her partner will be coordinated so as to facilitate participation and adherence to the study procedures. The family approach for initiation and follow up of HAART for women and partners will involve a specifically trained team of physicians, counselors, nurses and technicians from the departments of obstetrics, pediatrics, internal medicine and laboratory at each site.

Routine laboratory tests, including HIV ELISA, lymphocyte subsets, hematology and chemistries, will be performed in the study site laboratories, following the schedule shown in Table 1. The PHPT

laboratory and the Faculty of Associated Medical Sciences, Chiang Mai University will ensure training and quality control. Viral loads will be performed at the PHPT Laboratory. CD4 counts will be performed at selected hospital sites where quality and consistency of results have been assured and are monitored on a regular basis. Quality control for viral load testing will be performed on a monthly basis using an independent run control from the International Molecular Services (IMS). Every 3 months quality control for CD4 testing will use the proficiency panel from UKNEQAS [See appendix 7 for Laboratory Procedures]. Quality control for hospital hematology and chemistry laboratories is carried out through the Bureau of laboratory quality standard, Department of Medical Sciences, Ministry of Public Health.

C.3.1. Pre-enrollment Process

All HIV-positive women and /or partners identified at one of the 29 sites through the PMTCT program are offered counseling on PMTCT and HIV/AIDS and HIV confirmation serology and CD4 cell count measurement will be provided. At a subsequent visit, the internist will assess the need for OI prophylaxis and ARV treatment according to the MOPH guidelines for all identified immunocompromised patients. A special counseling session on ARV treatments will be offered to explain in detail the possible regimens, their constraints and potential side effects, and the importance of adherence. Patients with a CD4 count between **50** and 250 cells/mm^3 are eligible for the proposed study. They will be provided an appointment with a trained counselor to explain the study in details. All patients will be informed about the potential risks and benefits of the two monitoring strategies of ARV treatment in the study [See Section “Ethical considerations”]. Their participatory consent will be requested in writing. After consent, the baseline assessment for eligibility will be carried out [See Section C.2.2 for Eligibility, Inclusion and Exclusion Criteria]. A careful history of previous antiretroviral use will be taken. A clinical examination and baseline laboratory workup will be performed, including blood counts, renal, pancreatic and liver function, metabolic markers, e.g., glucose, cholesterol, and triglycerides, CPK, co-infection assessment (HBV, HCV), assessment of several infections (toxoplasmosis, syphilis, Mantoux test, chest radiograph, and CMV according to the Thai 2001 guidelines for the management of HIV infected patients), and HIV viral load measurement.

Women under ARV prophylaxis during pregnancy will be routinely followed monthly until the 34th week of pregnancy and then once every 2 weeks until delivery, according to the schedule recommended by the Ministry of Public Health. At delivery circumstances, mode of delivery, and any delivery complications will be recorded. Hospital staff will be specifically trained about confidentiality. Each site will maintain records of reasons for subject ineligibility or non-participation. As in the ongoing PHPT trial, special attention will be paid to needs of patients belonging to Thai minorities who may not be comfortable with standard Thai language (translation of documents, involvement of People with AIDS groups, availability of translators and training of counselors).

C.3. 2. Study Entry, Randomization

Patients will be given the results of laboratory tests performed at the previous visit. Counseling about OI prophylaxis and ARV treatments (in particular the first line regimen) will be reinforced. To ensure that patients are fully informed, they will receive in-depth information on the two monitoring strategies, treatment, and study schedule. Patients will be reminded that they can withdraw from the study at any time without compromising their future care. Partners may be randomized at this visit if they meet all selection criteria. Randomization of eligible pregnant women will take place, whenever possible, right after delivery when they are still hospitalized [See Section C.8 for Randomization Procedures]. ARV treatment will be initiated just after randomization. Patients will be given a one-month supply of antiretroviral treatment.

C.3.3. Follow-up

Study visits will take place **two** week after randomization and every month thereafter with a study nurse/counselor. Visits will include physical exam, counseling, adherence and tolerance evaluation, review of lab results and drug procurement. In addition, at 1 month and every 3

months thereafter until the end of the follow-up period, the physician will perform physical exam, assess HIV/AIDS related signs and symptoms as well as OIs and other infections, and review the blood tests performed within the previous month. These blood tests include: hematology, chemistry, CD4 counts, as well as drug dependent toxicity evaluations (SGOT, SGPT, alkaline phosphatase, bilirubin, creatinine, amylase, CPK, with triglycerides, cholesterol, and glucose measured every 6 months). A plasma sample will be also collected and stored frozen for viral load evaluation. For patients in the VL-S arm, real time viral load evaluation will be performed. For patients in the CD4-S arm reaching the CD4 switching criterion, viral load will be performed for final analysis. Patients may be seen more often as needed, in particular for toxicity, intolerance, HIV/OI related signs and symptoms or management of therapy. Other evaluations may be performed depending on the patient's condition.

Table 1. Summary of Clinical and Biological Study Follow-Up

	Screening period	Pre-Entry	Entry[1]/ Randomization	**2nd week**	1st month	Every month	Every 3 months
Questionnaire	X	X	X	X	X	X	X
Physical Exam	X	X	X	X	X	X	X
Counseling	X	X	X	X	X	X	X
HIV Serology	X	X					
Consent		X					
Adherence / tolerance to ARV				X	X	X	X
Hematology [2] Chemistry [3]		X			X		X
CD4+ cell count	X	X					X
Viral load [4]		X					X

1 Pregnant women may be randomized after delivery.
2 Hematology includes: Hemoglobin, hematocrit, RBC, MCV, WBC, platelets, reticulocyte count.
3 Chemistry includes: SGOT/SGPT, alkaline phosphatase, bilirubin, creatinine, amylase, CPK, triglycerides, cholesterol, glucose measured every 3 or 6 months.
4 Only the VL-S arm

Switching criteria and additional visits

In patients assigned to VL-S, after initiation of HAART (or any new regimen during the study), VL will be performed every three months. At month 3, if less than a ten-fold reduction in plasma HIV RNA is observed, patient adherence will be carefully re-assessed, and a new VL will be measured at 4 months. If there is still less than a ten-fold reduction in plasma HIV RNA (and if the HIV RNA is above **400 copies/mL**) the HAART regimen will be switched. Once a satisfactory VL has been reached (below **400 copies/mL**), if a viral load increase above this threshold of **400 copies/mL** is observed, a confirmatory viral load test will be performed within one month. Patients with a confirmed increase in viral load will be switched to second or third line treatment. In patients assigned to CD4-S, after initiation of HAART (or any new regimen during the study), switching will be performed when a confirmed (within one month) relative decline in CD4 count of more than 30% from baseline values **within 200 cells from baseline** is observed. After the first 6 months, if a 30% relative decline of CD4 counts from the "peak value" (the highest average of two consecutive CD4 counts) **within 200 cells from baseline** is observed, a confirmatory CD4 will be performed within one month. Patients with a confirmed decline in CD4 will be switched to second or third line treatment. In case of fever or intercurrent infections, CD4 and viral loads will be interpreted according to the clinical context and if necessary performed again when the condition has resolved.

If the CD4 decline or the VL increase is not confirmed, patients will continue the treatment and follow-up schedule. In all patients reaching the switching criteria, adherence and tolerance will be reassessed carefully before any decisions are made [See section C.4 for Adherence assessment and case management and section C.5. for Tolerance and safety assessment and case

management]. After treatment modification, a visit will be planned one week later to assess tolerance and adherence. Follow up will then resume with the original three monthly schedule of clinical and biological evaluation visits.

Before any confirmatory measurement (Viral load or CD4 count), patients will be instructed not to take their treatment the morning of the planned visit so that plasma can be stored frozen for retrospective analysis of drug trough levels. If needed the co-investigator can schedule extra visits/evaluations.

C.3.4 Repository of blood specimens

Samples, identified by code, will be stored in -70°C or -20°C freezers in the PHPT-IRD 054 repository building located in 31/1 Samlan Road Soi 1, Muang, Chiang Mai until the end of the study and only used for the study i.e. 6 months after all patients have completed the follow-up period which is the time necessary for laboratory tests to be performed and controlled and the results to be published. Patients can withdraw their blood sample from the sample repository at any time, and it will be immediately destroyed. No other studies will be carried out on these samples. If other studies were deemed necessary for a major scientific reason, we would request additional consent and this would be submitted as a specific study to the Ethical Review Committee for Research in Human Subjects.

The table below summarizes the quantity of blood drawn for each test.

Tests	Amount (ML)
HIV Serology	3 mL
Hematology	3 mL
Chemistry	3 mL
CD4+ cell count	4 mL
Viral load **and genotyping ***	6 mL

*** In the rare case where viral load would be too low to perform genotyping, another blood draw at a subsequent visit may be necessary.**

C.4. ADHERENCE ASSESSMENT AND CASE MANAGEMENT

We plan to implement a robust adherence assessment and management program to minimize the role of non-adherence as a possible cause of treatment failure before proceeding with switches. Assessments of adherence will provide feedback for how best to improve and/or maintain high adherence. Adherence will be assessed monthly using a combination of subjective and objective measures including pill count [151] and detailed self-report questionnaire [53, 151-155]. Adequate adherence will be defined as taking at least 95% of doses during each monitoring period. [C.7. Operational aspects of delivering HAART in Thailand; Appendix 8 for adherence assessment]

Counselors will be trained to develop culturally appropriate strategies to proactively assist and support patient efforts to adhere to the ARV regimens. They will meet individually with patients to provide a non-judgmental arena for information and support during ARV treatment. Counselors' responsibilities include: 1) patient education to explain the disease and treatment, purpose of therapy, medication regimens, management of side effects, the association of CD4 and viral load levels with treatment progress, risk of resistance, and importance of perfect adherence; 2) development of an individualized plan to integrate the regimen into patients' daily activities, including the use of medication boxes and personalized calendars as tools. Continuous reinforcement and follow-up will ensure optimal adherence.

Case management. Adherent patients will be encouraged to maintain their high levels of adherence. For non-adherent patients, the first step will be to reinforce adherence counseling. Counselors will target episodes of non-adherence by working with patients to devise strategies to prevent recurrent missed doses. However, if there is consensus between the physician, counselor, and patient that non-adherence is due to intolerance to a particular regimen, therapies will be modified, if possible, using drugs of the same class [See Section C.5 for Tolerance and safety assessment and case management].

For patients on a first line regimen who satisfy criteria for switching therapy and who are non-adherent despite counseling efforts, a second line regimen will be prescribed. If the patient remains non-adherent and reaches criteria for switching to a third line regimen, discontinuation of treatment will be considered [156]. Despite the decision to discontinue treatment, the patient will continue to be followed within the study. [See appendix 9 for side effects assessment]

C.5. TOLERANCE AND SAFETY ASSESSMENT

Management of toxicities will be discussed and reviewed with the clinical staff during on-site training that will be organized prior to starting the study. Additionally, these topics will be described in detail in the Manual of Operations [See Appendix 9 for Management of toxicities].

Safety and tolerance of therapy will be evaluated at each monthly visit. Laboratory results and clinical data will be evaluated every three months. If the patient presents with confirmed intolerance or side effects to one drug, that drug will be replaced by another drug in the same class**.** In case of intolerance, ZDV will be replaced by d4T, NVP by EFV, and Indinavir/Ritonavir by Nelfinavir.

Adverse events reporting. Severity of clinical and biologic toxicities will be evaluated according to standard assessment criteria that will be described in the Manual of On-site Operations [see Appendix 3 for PHPT-2 Manual of On-site Operations]. These are modeled after the toxicity tables developed by the ACTG.

Serious adverse events. Any serious adverse event and grade 4 toxicity will be immediately reported to the Study Coordinating Center (SCC), whether or not the event is considered to be related to ARV treatment. The SCC office can be reached 24 hours a day by a paging system and phone. All these events will be reported within 48 hours to the Ministry of Public Health and to the drug manufacturers. The form for reporting adverse events is provided in Appendix 4: Serious Adverse Event Form.

C.6. ASSESSMENT OF RESISTANCE

Currently, two types of HIV resistance analyses are available: phenotypic and genotypic testing. Phenotypic testing assess the ability of specific drugs to inhibit viral replication in cultured cells, but are restricted to specialized laboratories and commercial companies due to their time-consuming and costly nature. Genotypic resistance tests assess the genetic composition of the reverse transcriptase and protease genes using PCR technology. Following amplification, the genes are subjected to automated DNA sequencing to detect any mutations (Visible Genetics, ABI/Perkin-Elmer, Affymetrix) or point mutation assays (Innogetics and Chiron).

The relative simplicity and reliability of the genotypic assay used to determine drug-related mutations makes it more feasible than any of the phenotypic assays for initial, real-time measurement of resistance in this study. Several laboratories in Thailand have expertise in genotypic resistance testing and were trained by our group at Harvard (Chulalongkorn and Mahidol Universities). These tests are presently done for research purposes only. We anticipate that, although real time resistance testing will not be feasible in this trial and is not likely to become part of the standard care in Thailand in the short term, for safety reasons batched genotypic resistance testing will be performed both in VL-S subjects who satisfy switching criteria because of virologic failure and in CD4-S subjects who satisfy switching criteria because of CD4 cell loss.

New mutations associated with drug resistance are being discovered regularly. Exhaustive and regularly updated resistance patterns to antiretroviral drugs are made available by the International

AIDS Society-USA [57] (www.IASUSA.org). Resistance interpretation is complex and several groups provide regularly updated algorithms for analysis, including the Stanford HIV RT & Protease Sequence database [9] (hivdb.stanford.edu).

Identification of primary and secondary protease and RT mutations will be carried out using the regularly updated consensus tables and algorithms from the International AIDS Society – USA Drug Resistance Mutations Group as well as Los Alamos and Stanford.

In subgroup B strains, ZDV resistance emerges slowly over months/years through the selection and accumulation of mutations at many different RT codons [157]. The most important RT codon mutations cause substitutions at amino acids 41, 67, 70, 215, and 219. M184V causes high level (>100-fold) 3TC resistance. It emerges rapidly in patients receiving 3TC monotherapy and is usually the first mutation to develop in a failing 3TC-containing regimen. The multinucleoside resistance Q151M mutation confers intermediate levels of resistance to ZDV, ddI, d4T and ABC. It is generally followed by mutations at codons 62, 75, 77 and 116, which induce high-level resistance to each of these NRTIs. For NNRTI a single mutation is enough to confer high-level drug resistance. K103N is the most clinically important NNRTI resistance mutation as it is sufficient to cause virological failure with any of the three existing NNRTI. The V106A mutation causes >30 fold resistance to NVP, intermediate resistance to DLV and low level of resistance to EFV. Resistance to PIs develops in a stepwise manner through the accumulation of mutations in the HIV-1 protease gene [119, 120]. Primary PI mutations (such as G48V, V82A/T/F/S, M46I/L D30N, I84V and L90M) decreased binding of the PI to the protease and are generally the first to be selected. Secondary mutations can be compensatory to the primary mutation to improve viral fitness.

Because the systematic study of both genotypic and phenotypic data on clinical isolates from large numbers of patients followed with carefully collected clinical data can reveal unsuspected patterns of mutations, we plan to collaborate with commercial companies to study the phenotypic HIV resistance patterns. This study will be designed and implemented as a separate, but connected project related to the trial [See Appendix 11, Letters of Collaboration]. In addition, it will be important for the interpretation of the study results to evaluate viral replication capacity (fitness), especially in the group under CD4-S [134]. This will be done in a retrospective fashion, on stored frozen samples, comparing replication capacity at base line and at times of peak immunological response and immunological failure. As with the investigation of phenotypic resistance, collaborations will be sought, and it is likely that additional funding will be required.

Sequencing of RT and protease genes will be performed using the HIV-1 Genotyping Systems kit (Applied Biosystems, Foster City, California, USA), according to the manufacturer’s guidelines. Experience in related projects (the U.S. ACTG and the World Health Organization (WHO) project for global surveillance of resistance) suggests that it is important to analyze and quality-control genotypic data in ways going beyond the checks built into the ABI system. One needs to determine the starting point of the sequence generated by lab equipment in each of the two genes of interest; to detect, document, and correct for frameshifts; to align the sequence in a way accounting for (and documenting) insertions and deletions; to detect illegal nucleotide codes and stop codons (both of which indicate quality problems in the sequence); to check for unusually high frequencies of mixture or ambiguous codes; to analyze sequences for signs of contamination; and to examine mutational patterns, double-checking abnormal results. Since subtypes vary, it is helpful to examine mutations in relationship to a reference wild type sequence that is of the same subtype as the one under analysis, and this implies determining which subtype that sequence belongs to (although the determination is necessarily approximate when only protease and RT data are available).

Frontier Science and Technology Research Foundation provides data management (and through the Statistical Data Analysis Center located at Harvard, statistical analysis) for the ACTG. It is also the Data Center for the WHO global surveillance project, and in that role has been analyzing and organizing genotypic data from labs in Brazil, France, Israel, South Africa, Thailand, and Uganda. It has a comprehensive, web-based system for analyzing sequences, implementing the quality checks mentioned above, and will make that system available to this project, generating reports on each sequence.

In addition, we will include a quality assurance program similar to the Virology Quality Assurance systems in the ACTG. We will submit to our colleagues at Frontier Science the sequence data

provided by the reference laboratory as well as those generated by our own laboratory to compare reports and make sure that our laboratory generates consistent results.

C.7. COHORT RETENTION

Patients will be followed at the hospital the family chose for the follow-up of the pregnancy, care of the child and HIV treatment. The process of informed consent counseling which involves explanation of the study objectives, potential risks and benefits before enrollment, as well as the ongoing counseling at each visit will be designed to ensure that patients understand the necessity for them to comply with the protocol requirements. Moreover, the family approach to HIV care and treatment is expected to facilitate adherence to the protocol.

As in previous PHPT studies, patients are asked at each visit whether they have moved since their last visit or plan to move. In cases of missed scheduled appointments, the patients will be contacted by mail or phone, or visited by a social worker. In addition, patients will have access to the study clinic for counseling and care at any time throughout the study period and can reach the nurse in charge of their follow-up by telephone. When appropriate, patients can be reimbursed for transportation and medication costs. The DSMB will review enrollment, compliance, and study retention and make recommendations as appropriate.

The study nurse will see patients every month for drug dispensation and adherence/tolerance evaluation and the physician every 3 months for clinical and biological evaluations. It is our experience that such frequent patient/medical team contact is very well accepted and essential for improving adherence, providing ongoing counseling and information, and preventing toxicities.

C.8. RANDOMIZATION PROCEDURES

We anticipate that 60% of the patients enrolled will be women. The study statistician will generate two randomization lists per site so that mothers and their partners are randomized separately.

Each list, (by blocks of 4 to 8, depending on the expected site enrollment) will be composed of running randomization numbers. For example, each block of 8 numbers will include 4 numbers associated with VL-S and 4 associated with CD4-S. Block size will not be disclosed to the study site so that at no time a co-investigator can guess what will be the monitoring scheme of the next patient.

As blinding in this trial is not feasible, **randomization** will be centralized and it **will take place after the patient has been enrolled and the antiretroviral treatment has been started. Since the first treatment regimen is the same in both study arms, the investigator will not have to wait for randomization to start treatment When the enrollment has taken place, the co-investigator will send a Declaration of new enrollment form to the Study Coordination Center specifying that the patient started the first line regimen.** The Study Coordination Center will refer to the lists of randomized numbers for the site, assign the patient to the next number, and send a fax to the co-investigator specifying the patient number and the corresponding monitoring scheme.
Case Report Forms for each study visit will be provided for each patient. Study participants will not be identified by name on any study documents but will be identified by a Patient Identification Number. All laboratory specimens, evaluation forms and reports will only be identified by a coded number. All records will be kept in a locked file cabinet in the clinical research unit. All computer entry and networking programs will only be processed with coded numbers. Clinical information will not be released without the written permission of the patient.

C.9. STUDY DRUG SUPPLY, DISTRIBUTION AND STORAGE

Antiretroviral drugs for this trial will be supplied by the manufacturers in the form of study supplies and will be distributed to the study sites' clinical co-investigators/pharmacists through the central pharmacy in Chiang Mai. The study pharmacist at the SCC supervises the management of stock, storage, dispatch of the study drugs, and ensures that they are properly pre-packaged and pre-labeled. Study drugs will be stored in a locked cabinet devoted specifically to the study under the

responsibility of the hospital pharmacist. Each site pharmacist will maintain records of all study drugs received and their storage conditions. Pharmacists will return all unused or undispensed study drugs to the Chiang Mai study coordination center.

C.10. STUDY MANAGEMENT

All study monitors, data managers and clinical co-investigators have received training for human subject protection.

The Study Coordination Center (SCC) team is responsible for the overall organization and monitoring of the study, in particular compliance with Good Clinical Practices (GCP) [158] and reporting to the sponsors, as well as data entry, data management and statistical analysis. Site co-investigators are responsible for the medical management of the patients. Routine laboratory exams and procedures necessary for enrollment and follow-up of patients are done at each site. The PHPT laboratory is responsible for the centralized laboratory exams, viral loads, resistance testing, and samples repository. Quality control of the site laboratories is ensured in collaboration with the Faculty of Associated Medical Sciences, Chiang Mai University.

The Study Coordination Center staff for PHPT includes a coordinator, a pharmacist, two clinical safety monitors, ten clinical research assistants, three data managers, four data entry technicians, five laboratory technicians, two logisticians, a translator and three secretaries. This team is well trained in the management of clinical trials in HIV/AIDS and their roles have been refined through the course of the PHPT studies. They are highly motivated and will be carried over to manage the proposed study and adapt the procedures developed for PHPT-1 and PHPT-2 to the new protocol.

The study teams at each site will consist of physicians, nurses, laboratory technician, pharmacist and counselors, in addition to the investigators. Training of all research team members will take place in the initial period of the study, and will include sessions on HIV management and antiretroviral therapy, counseling, confidentiality, compliance and procedures designed for study implementation. This training has been proven useful to ensure compliance with GCP and to minimize loss to follow-up.

Prior to the beginning the study, the manual of operation will have been translated into Thai and reviewed by all research team members. One training session will focus on the importance of high quality data collection and compliance to the protocol on the final study results. A bi-monthly newsletter/report will be used to highlight and further clarify these issues.

Every form will pass through the following steps: completion; on-site checking by the clinical research assistant (CRA); tracking at SCC; review by the safety manager at SCC and investigation if necessary; double data entry; file comparison; and 100% verification.

At their weekly visits, the CRA, under the supervision of the SCC, will monitor the quality and accuracy of data collected in the research records, and the concordance with source documents, and will check that all regulatory and clinical safety requirements are met. The data management team at the SCC will review all forms for completeness and any discrepant responses. In the case of incoherent or missing data, the study site team will be immediately contacted and, if necessary, the form returned to them for correction. The CRA will be responsible for obtaining answers to queries during the weekly site visits.

The data entry and management programs, written by PHPT study statisticians in charge of data management, will be adapted for this proposed study. These programs check for duplicates and examine the dataset for range checks on each field within a form, consistency checks between fields on the same form, and between fields on different forms. All forms coming from the sites are recorded and filed upon arrival at the SCC. The tracking database in place at the SCC will provide updated information on each patient every week, as well as the total number of patients at each stage of follow-up. A program generates the list of missing pages/forms.

The data managers generate queries until final validation of the CRFs. Validated CRFs serve as evidence that the history and clinical data has been recorded, and that all lab tests and procedures have been completed. An audit trail is maintained for all changes made to the original data set as required by GCP. There will be ongoing monitoring of the safety profile of each patient by the SCC

and all serious adverse events will be reported according to GCP. (Regular meetings will be organized with clinical investigators and their teams to review the study monthly reports).

C.11. MAIN ENDPOINTS

The primary endpoint is clinical failure defined as the occurrence of confirmed CD4 below 50, first or new AIDS-defining event, or death. The main secondary endpoint, related to the preservation of treatment options, is the number of drugs exhausted, taking into account cross-resistance mutations.

The quantitative measure of the therapeutic options left at a given time as determined by mutation pattern of the genotypic sequence of subject-patient derived virus will use the metric developed by Dr. V. De Gruttola and H. Y. Jeing for use in protocol A5115, denoted FD. First, the interpreting system for genotypic sequence data "Stanford Inferred Drug Resistance (beta test)" is used to determine which drugs the patient-derived virus is sensitive to. Then the number of classes of drugs that include at least one drug to which a patient's virus is sensitive is counted and denoted NC. If a subject's virus is sensitive to all drugs in NRTI class, 0.3 credit to NC is added; similarly, 0.3 credit to NC for full PI class sensitivity is added. The resulting score is the value of FD.

Note: Additional information will be generated and provided to the Resistance Experts Committee in order to complete the resistance data and cross check coherence with FD: the total genotypic sensitivity score (GSS) computed by adding, for each drug the patient is receiving, 1 if there is sensitivity or 0 if there is resistance; and the total number of mutations per the IAS-USA table, and the patient's treatment history.

The secondary endpoint related to safety will be time to the first development of grade 3 or grade 4 sign, symptom, and laboratory abnormality. For secondary analysis, endpoints related to virological and immunological failure are defined as for switching criteria: VL above **400 copies** per mL and 30% CD4 decline from observed peak values **within 200 cells from baseline. Note: failure to raise CD4 by more than 50 cells after one year of therapy in the presence of viral replication would be considered as an immunological failure. Similarly, failure to decrease VL below 400 copies by 6 months would be considered as virological failure. In both cases, the "Resistance Experts Committee" would review the patients' data and would advise on the most appropriate course of action for these patients.**

C.12. STATISTICAL CONSIDERATIONS

C.12.1 General design issues.

The proposed study is designed primarily to test the non-inferiority of the CD4 based monitoring strategy compared to the standard viral load based strategy. To be more precise, we would like to demonstrate that, given the expected advantages of using a simplified CD4 based monitoring strategy; this strategy does not lead to an unacceptably higher risk of clinical failure compared to the standard monitoring strategy.

A very important secondary objective is to determine whether or not there is a benefit in terms of drugs spared, taking cross-resistance mutations and cross-toxicities into account, when using CD4-S instead of VL-S.

C.12.2 Power and Sample Size.

The study will test for non-inferiority between strategies, i.e. that at 3 years the risk of clinical failure under CD4-S (P_{CD4}) is <u>not higher</u> than the risk of failure under VL-S (P_{VL}) by more than a predefined difference, delta, considered clinically acceptable given the expected benefit of using the alternative strategy. CD4-S will be considered non inferior to VL-S for practical purposes if the upper limit of the confidence interval of the difference between P_{CD4} and P_{VL} failure rates is smaller than delta [159].

The null hypothesis is that the difference of clinical failure rates between the CD4-S and VL-S arms is equal to or greater than delta (H0: $P_{CD4} – P_{VL} \geq$ delta) [159, 160]. The alternative hypothesis is that failure rate under CD4-S relative to VL-S is inferior to delta (H1: P_{CD4}-P_{VL}< delta).

The first type of error that we are concerned about is the declaration of non-inferiority of CD4-S compared to VL-S when the true difference in failure rates is equal to or greater than delta. Therefore, the non-inferiority test will be designed so that this risk is no more than 5%. Another potential class of error would be to conclude that the two monitoring schemes are not equivalent when the true difference between CD4-S and VL-S is less than delta. This would result in choosing to keep VL-S when the alternative CD4-S is not inferior. In this trial, the sample size will be such that the risk of missed non-inferiority is less than 20%, assuming $P_{CD4} = P_{VL}$.

For calculation of the sample size, a value for P_{VL} must be assumed and an acceptable difference delta must be defined. Based on the review of the literature [11, 129, 130, 132, 161], we have hypothesized that in a population of ARV naïve patients **without symptoms** initiating HAART with **CD4 count between 50** and 250, the risk per year of clinical failure under VL-S would be 5%, i.e. P_{VL} approximately 14% after 3 years. With a value for delta set to 7.4%, which corresponds to a hazard ratio (Rate of failure under CD4-S relative to the rate of failure under VL-S) of 1.6, the sample size ensuring 80% power to detect non-inferiority of CD4-S is 304 evaluable patients.

Because we are mostly interested by the comparison of P_{CD4} and P_{VL} at three years when we will also be able to compare the strategies in terms of treatment options preserved, we have presented a non-inferiority test based on an absolute difference delta between failure rates. However, the fixed hazard ratio 1.6 will be used to determine the critical limit for non-inferiority testing for two important reasons. Firstly, use of a hazard ratio will be necessary for comparing clinical outcomes at interim analysis; secondly, a constant delta does not have the same meaning at different levels of clinical failure rates (Table 3). For example, a fixed difference of 8% represents a several fold risk increase when P_{VL} is in the 1 - 2% range, while the same difference can become acceptable when P_{VL} is higher than 10%.

In order to investigate the consequences of a variation of P_{VL} around the expected value of 14%, we have calculated the sample size for different values of $P_{VL,}$ hazard ratios (and corresponding delta), and power levels (table 3).

Table 3: Number of evaluable patients per arm necessary to guarantee a 80 or 90% power in a one-sided non-inferiority test, according to P_{VL} and the delta difference, corresponding to the predetermined hazard ratio.

3 year VL-S Clinical Failure rate		10%	11%	12%	13%	14%	15%	16%	17%	18%	19%
Hazard Ratio 1.5	Delta	0.046	0.050	0.054	0.059	0.062	0.066	0.070	0.074	0.077	0.081
Power	90%	800	729	670	621	578	541	509	481	456	433
Power	80%	587	535	492	455	424	397	373	352	334	317
Hazard Ratio 1.6	Delta	0.055	0.060	0.065	0.070	0.074	0.079	0.083	0.088	0.092	0.096
Power	90%	571	521	479	444	413	387	364	344	327	311
Power	80%	420	383	352	326	304	284	268	253	239	228
Hazard Ratio 1.7	Delta	0.064	0.070	0.075	0.081	0.086	0.091	0.097	0.101	0.106	0.111
Power	90%	430	393	361	355	312	293	276	261	247	235
Power	80%	318	290	277	249	230	215	203	192	182	173

To meet the study objectives for the primary hypothesis, 608 evaluable patients or 700 enrolled patients assuming that 15% of the patients will not be evaluable, will be sufficient. However, based on the number of events observed at each interim analysis, the DSMB may recommend an adjustment of the sample size or of the duration of follow up.

Table 4 shows the results of testing for non-inferiority of CD4-S versus VL-S with 304 evaluable patients in each arm, a hazard ratio of 1.6 (delta .074), a value for $P_{VL}$of 0.14 and various values of P_{CD4}.

3 year VL-S Clinical Failure rate P_{VL}	.1400	.1400	.1400	.1400	.1400
3 year CD4-S Clinical Failure rate P_{CD4}	.1600	.1625	.165	.1675	.17
95%CI Upper boundary of the difference of failure rates	.0677	.0700	.0730	.0757	.0783
Conclusion of CD4-S non inferiority	No	No	No	Yes	Yes

With 1.6 as critical hazard ratio for non-inferiority and a 14% observed P_{VL}, we see that CD4-S will be declared non inferior statistically with P_{CD4} equal to 16.5%, but will be declared inferior with P_{CD4} equal to 16.75%.

C.12.3 Data Monitoring

As was done in PHPT-1 and -2, screening, accrual and safety data will be reviewed by the monitoring team every week. The protocol statistician will regularly report the results to the study team. A monthly study report with screening, accrual, and patient characteristics at entry and at each subsequent visit, as well as safety data (blind to randomized treatment assignments), will be issued throughout the duration of the study.

C.12.4 Interim Monitoring

This study will be monitored regularly for toxicity, compliance, and feasibility. Two interim efficacy analyses and one final analysis are planned. Monitoring of clinical failure rates will be conducted for patient safety. The trial will be discontinued if the clinical failure rate in the CD4-S group is clearly higher than in the VL-S group.

The interim analysis will be based on comparison of the time to clinical failure curves using Kaplan-Meier estimates and log rank statistics (one sided). For stopping rules, O'Brien-Fleming boundaries [162] will be used to adjust for the multiple looks, as modified by Lan and Demets [163]. The two interim analyses will be conducted when approximately 33% and 67% of the expected number of clinical failures will have occurred. The Z statistics for stopping the study and final analysis are 2.9611, 2.0938, and 1.7096.

Therapeutic options left to patients according to the pattern of resistance acquired under each of the monitoring strategies is the most important secondary endpoint of this study and will be assessed for safety purposes at each interim analysis. Due to the complexity and rapid evolution of the field, genotyping information will be reviewed prior to interim review by the study statistician and a group of experts in resistance mutation testing/interpretation. At least one member of the DSMB will be a specialist in resistance mutations. Patterns of resistance mutations, number of drugs and classes used, as well as number of drugs remaining available based on the IAS-USA interpretation tables will be compared between groups. The metrics developed at Harvard will greatly facilitate this comparison as it quantifies, in a single measurement, the expected therapeutic options left for patients at any given time point which can then be analyzed using standard statistical methods [See Section C.13.Data and Safety Monitoring Board below].

The quantitative measure of future drug options (FD) based on mutational resistance pattern of patients derived virus is an ordered categorical variable which can take values of 0, 1, 1.3, 2, 2.3, 2.6, 3, 3.3, or 3.6 if the three possible drug classes (NRTI, NNRTI, PI) are considered.

Our colleagues at the HSPH Department of Biostatistics performed an exploratory analysis using genotypic data from ACTG 364, ACTG 372, ACTG 359, and ACTG 398 to investigate the properties of this endpoint in view of its utilization in protocol ACTG5115. The estimates of standard deviation of FD among HAART-experienced patient subjects appear highly consistent across the studies. The pooled data gives an estimate of standard deviation of FD 0.99, which can be assumed as the maximal standard deviation of FD in our study where all patients being antiretroviral naïve are assumed to present uniformly with the maximum score at study entry. At each interim analysis FD will be compared across the two strategy arms using a chi square test for ordinal categorical data with a two-sided 0.05 significance level [164]. For those who are undetectable at the time of analysis we will use their last available genotypic testing, or the assumed maximum score at baseline. A one point deviation of the median FD would be considered of major clinical importance at the population level and would trigger an extensive review of the laboratory data by the resistance committee including patterns of virological (% above **400 copies/mL**) and immunological failures (% with CD4 decline more than 30% **within 200 cells from baseline**), patterns resistance/cross resistance and drug toxicities/intolerance.

C.12.5 Analysis Plan

All analysis will primarily follow the intent to treat philosophy. However, per protocol comparisons will also be performed in order to take into account protocol deviations and cohort retention.

Primary analysis will compare the group randomized to CD4-S with the group randomized to VL-S based on the Kaplan-Meier estimates of the clinical failure rates at 36 months and their Greenwood standard error estimates. As suggested by Makuch and Simon [165], monitoring scheme comparisons will be based on the confidence interval for the difference between the treatment failure rates. The upper 95% one-sided confidence boundary for the true difference in failure rates between CD4-S and VL-S will be calculated. If this confidence boundary is less than the threshold value delta corresponding to a 1.6 hazard ratio, this will be taken as evidence that, from a clinical perspective, CD4-S is not inferior to VL-S. In this case, we would be 95% confident that the failure rate under CD4-S is not higher than the failure rate under VL-S by more than delta.

In a secondary analysis, confidence intervals of the failure rates will be provided to assess consistency of the overall result within patient stratum (women initiating therapy after delivery or partners).

Time-to-event methods, including log rank tests, and Kaplan-Meier cumulative event curves will be used to summarize the major outcomes of new or recurrent disease progression, including death, and CD4 decline below 50.

Although randomization will be the first step to balance known and unknown covariates between study arms, we will compare the distribution of all baseline characteristics among arms (chi square and Fisher's exact test when appropriate for qualitative variables, Wilcoxon for continuous variables). The analysis will be stratified on factors associated with the outcome such as clinical stage of disease, CD4 count, and viral load. For time to event outcomes, proportional hazards (Cox) models will be used to adjust statistical inferences for these factors.

The most important secondary objective of this study is to evaluate the impact of switch strategies on viral resistance by comparing a quantitative measure of future drug options as determined by genotypic sequence data obtained across the two strategy arms. This comparison will use the specific metric developed by V. de Gruttola and H. Y. Jeing, Harvard School of Public Health as described in Section C.11 Main Endpoints and in Section C.13 Data and Safety Monitoring Board [164].

Other comparisons related to the preservation of treatment options between monitoring strategies will include the number of drugs used, taking into account cross-resistance mutations and cross toxicities; time to first switch and number of switches over the first 36 months follow-up period as well as the proportion of patients still on initial therapy.

Time to first therapy change, virological and immunological failure under first line therapy will be studied in each arm using proportional hazard model (Cox) taking into account covariates such as viral load and CD4 count at entry, and adherence. This will allow the investigation of the role of baseline plasma HIV-1 RNA and CD4 measurements as predictors of treatment modifications, virological and immunological failure as well as the degree to which toxicity, noncompliance and viral genotype (treated like prognostic factors) also contribute to treatment modifications, virological and immunological failure.

Depending on the number of drugs to which patients will be exposed, one could expect a difference in safety profile in relation to monitoring scheme. Clinical laboratory data and adverse experiences will be examined and will include plots of the data as well as summary statistics of the raw data and changes from baseline. Wilcoxon rank sum tests will be used to compare the groups for changes from baseline. The proportion of patients in each arm who experience toxicity will be compared using a two-sided Fisher's exact test. The proportion of patients in each arm reporting each individual type of adverse experience will be calculated. Differences in the severity of toxicities between groups will be evaluated, with each type tested at the P=0.05 two -sided level of significance. Due to multiple toxicity types, this evaluation will not represent a formal statistical test of a specific hypothesis.

Each of the statistical analyses on secondary endpoints will be conducted at the nominal .05 level of significance without adjustment for multiple comparisons.

C.13. DATA AND SAFETY MONITORING BOARD

Resistance Experts Committee. For patients assigned to the experimental arm (CD4-S), there is a potential for increased risk of resistance and cross-resistance, theoretically leading to fewer treatment options. For this reason, a Resistance Experts Committee will be constituted [See Appendix 11, Letters of Collaboration, for the members of this committee] consisting of experts in resistance mutations from USA, Thailand and Europe. In order to track this problem, resistance mutations will be assessed in all patients with viral load > **400 copies/mL** in the VL-S arm, all patients who fulfill criteria for switching in the CD4-S arm, plus a random sample of other subjects in the CD4-S arm so that measurable and comparable data can be analyzed from equivalent numbers in the two arms. This will be done once a year and more often if deemed necessary by the DSMB. The Resistance Experts Committee will work independently from the research team and will provide expertise in support of the DSMB regarding the development of resistance mutations and their clinical implications. The Committee will be chaired by a member of the DSMB. Members of the Committee can be, but are not required to be, members of the DSMB because of logistical complexity. Members of the Committee will review closed interim reports, and the Chair of the Committee will have the responsibility to write the summary report of the Resistance Experts Committee deliberations and present that summary to the DSMB. The Resistance Committee does not make recommendations about the conduct of the study. In addition, depending on the specific patients' treatment regimen and their pattern of virological/immunological response, individual **clinical, virological, immunological and** genotyping results may be submitted on an ongoing basis for individual safety purpose, in order to avoid an excessive accumulation of resistance mutations and to be able to modify therapy in a timely manner. It is anticipated that such case will be rare and will not affect the relevance of the study.

Patterns of resistance mutations, number of drugs and classes used, as well as number of available drugs remaining based on the IAS-USA interpretation tables will be compared between groups by the study statistician. The committee will be asked to provide expert judgment according to current knowledge, including the criteria and methods developed by Dr. Victor de Gruttola at Harvard. If it appears that the patients enrolled in the CD4-S arm have a resistance pattern that significantly jeopardizes their future treatment options compared to patients in the VL-S arm, the DSMB will be advised of this and a DSMB meeting will be convened to provide further recommendations with regards to the conduct of the study. The resistance committee will meet at least every year or more often if deemed necessary. At the end of year three, should the committee conclude that a longer follow-up is necessary, the investigator will seek additional funding for an extension of the follow-up.

Data Safety and Monitoring Board: The Data and Safety Monitoring Board (DSMB) has been specifically asked to evaluate safety and study parameters such as enrollment, compliance, follow-up, laboratory evaluations, data submission, and quality control every six months throughout the course of the study. The DSMB will advise at each of these reviews whether the study should continue as originally designed. Efficacy analyses will be conducted for two interims and one final evaluation. The interim analyses will be presented to the DSMB when 33% and 67% of the expected number of clinical failure will have occurred. The DSMB will consist of five members, three of whom will be Thai. Three will be physicians and two statisticians, with at least one specialist of genotypic resistance mutations interpretation (the Chair of the Resistance Experts Committee). None of the DSMB members will be directly involved in the conduct of the study. All meetings will take place in Thailand. A DSMB composed of five members, three of them Thais, has overseen the conduct of each of the two previous PHPT studies. These groups have functioned efficiently and have successfully played their role as an independent body, advising the research team on the best interest of the patients and the best conduct of the study.

C. 14 PHARMACOKINETIC SUB-STUDY

The second line HAART regimen in this study contains IDV/RTV, bid. The pharmacokinetics of ZDV, ddI, and 3TC are linear within the range of dosing used in clinical practice. The

pharmacokinetics of zidovudine (ZDV) has been investigated in both Caucasians and Thais and no marked ethnic difference in ZDV phosphorylation was observed. 3TC and ddI have been used in Thailand and no unexpected toxicity was reported. The pharmacokinetics of IDV, in contrast, is not linear. With 800 mg tid dosing, it has been found to be similar in Thai and Caucasian patients. Safety and efficacy of IDV (800 mg, tid) versus IDV/RTV (800 mg/100 mg, bid) were found to be equivalent in both European and Thai populations, although there was a slight trend towards more adverse events in the bid arm in Thai patients. Indeed, the trough concentrations (Cmin) of IDV/RTV 800mg/100mg bid are significantly higher than in IDV 800 mg tid (1,400 ng/ml and 130 ng/ml respectively), and the AUC is two fold higher in the bid regimen. Recently, IDV/RTV (400 mg/100 mg, bid) has been reported to be well tolerated, with median IDV concentrations at day 15 falling within the therapeutic range (Trough levels higher, peak level lower, AUC equivalent).

To assess the inter-individual variability of drug levels, data will be collected in the first 20 patients participating in the randomized study and consenting for this substudy. In these patients, blood samples will be drawn one and two months after initiating the first line HAART regimen at predose and 1, 2.5, 4 and 12 h after ingestion of the study treatment.

For each drug at each dosing, we will estimate the trough concentration (Cmin), time to maximum concentration (Tmax), maximum drug concentration (Cmax), elimination half-life (T1/2), area under the concentration time curve (AUC) and oral clearance (CL/F). We will estimate all the PK parameters using a population approach. Pharmacokinetic calculations will be performed using NONMEM V.

To relate drug exposure (adherence, pharmacology) to the patient's outcome, we will assess the plasma antiretroviral drug levels after one month of a new therapy in blood samples drawn for the regular follow-up of the patients. The assessment of plasma drug levels can also be performed in other samples, drawn at any time for the immunological and virological follow up.

C.15. ARV MANAGEMENT STRATEGY FOR IMPLEMENTATION IN THE THAI CONTEXT

An important public health objective of this study is to document HAART monitoring strategies in order to help their implementation in the Thai context, more specifically within the mother and child health framework.

This trial, while testing two monitoring strategies, one simpler than the other, will at the same time involve, in both arms, intense and frequent clinical assessments as well as complex retrospective analysis (e.g. viral fitness assays and genotypic resistance mutations). We realize that these labor-intensive components of the trial will not, in all likelihood, be directly applicable as standard of care. Yet, this trial will provide the necessary empirical evidence for designing future monitoring schemes appropriate to the various Thai contexts. The results and, more generally, the data generated in this trial will have to be put into practice, in particular with regard to the optimal frequency of evaluations, the relative importance of clinical, CD4 and viral load monitoring, the screening and management of metabolic/hematological toxicities and intolerance, the assessment/reinforcement of adherence, and the role of couple counseling for improved patient management.

Investigating implementation of HAART in the Thai context is critically important as it coincides with the MOPH officially launching its new program "Expanding HIV care or treatment for Families" (Division of AIDS, Department of Communicable Diseases Control and Bureau of Health Promotion, Department of Health, December 21, 2001). Along with PHPT, colleagues from the Thai-US CDC collaboration, the MOPH Departments of Communicable Diseases Control and Health and Provincial Health Offices participated in the first workshop for the development of this program. PHPT was specifically asked to help design a model for the delivery of HAART therapy within the existing Mother and Child Health framework. The year 2002-3 development plan includes the assessment of the clinical, laboratory and psychosocial infrastructure of the program, the review of adult and pediatric OI and HIV treatment guidelines as well as the establishment of a consensus on the management of asymptomatic and symptomatic women, partners, and children.

A training and capacity building program will be initiated and appropriate monitoring and evaluation tools developed.

We will collaborate with the Ministry of Public Health, in particular its Health Systems Research Institute, to develop the framework for data collection and analysis at the hospital level that will help shape policy decisions regarding HAART management strategies.

C.16. STUDY TIMETABLE

Table 5. Study timetable

Month of Study	-6	0	6	12	18	24	30	36	42	48	54	60	
Study preparation (Manual of Operation and CRFs, study drug, sites)	▸												
Randomization		▸	▸	▸	▸	▸							
Patients on study drug		▸	▸	▸	▸	▸	▸	▸	▸	▸	▸	▸	
Last patient visit								▸	▸	▸	▸	▸	
Virological Studies			▸	▸	▸	▸	▸	▸	▸	▸	▸	▸	
Data Management			▸	▸	▸	▸	▸	▸	▸	▸	▸	▸	▸
Interim and Final analyses				▸			▸					▸	

D. LITERATURE CITED

1. Erali M., Page S., Reimer L. G. and Hillyard D. R.: Human immunodeficiency virus type 1 drug resistance testing: a comparison of three sequence-based methods. J Clin Microbiol 2001; 39:2157-65.

2. Schuurman R., Brambilla D., de Groot T., Huang D., Land S., Bremer J., Benders I. and Boucher C. A.: Underestimation of HIV type 1 drug resistance mutations: results from the ENVA-2 genotyping proficiency program. AIDS Res Hum Retroviruses 2002; 18:243-8.

3. Wensing A. M. J., Keulen W., Buimer M., Brambilla D., Schuurman R. and Boucher C.: The ENVA-3 world wide evaluation study shows extensive differences in interpretation on HIV-1 genotype analysis. 41 st Intersciencw Conference on Antimicrobial Agents and Chemotherapy, 2001.

4. Fontaine E., Riva C., Peeters M., Schmit J. C., Delaporte E., Van Laethem K., Van Vaerenbergh K., Snoeck J., Van Wijngaerden E., De Clercq E., Van Ranst M. and Vandamme A. M.: Evaluation of two commercial kits for the detection of genotypic drug resistance on a panel of HIV type 1 subtypes A through J. J Acquir Immune Defic Syndr 2001; 28:254-8.

5. Mukaide M., Sugiura W., Matuda M., Usuku S., Noguchi Y., Suzuki K., Kawata K., Ito A., Sagara H., Yamada K., Kondo M. and Imai M.: Evaluation of Viroseq-HIV version 2 for HIV drug resistance. Jpn J Infect Dis 2000; 53:203-5.

6. Mracna M., Becker-Pergola G., Dileanis J., Guay L. A., Cunningham S., Jackson J. B. and Eshleman S. H.: Performance of Applied Biosystems ViroSeq HIV-1 Genotyping System for sequence-based analysis of non-subtype B human immunodeficiency virus type 1 from Uganda. J Clin Microbiol 2001; 39:4323-7.

7. Cunningham S., Ank B., Lewis D., Lu W., Wantman M., Dileanis J. A., Jackson J. B., Palumbo P., Krogstad P. and Eshleman S. H.: Performance of the applied biosystems ViroSeq human immunodeficiency virus type 1 (HIV-1) genotyping system for sequence-based analysis of HIV-1 in pediatric plasma samples. J Clin Microbiol 2001; 39:1254-7.

8. Eshleman S. H., Gonzales M. J., Becker-Pergola G., Cunningham S. C., Guay L. A., Jackson J. B. and Shafer R. W.: Identification of Ugandan HIV type 1 variants with unique patterns of recombination in pol involving subtypes A and D. AIDS Res Hum Retroviruses 2002; 18:507-11.

9. Kantor R., Machekano R., Gonzales M. J., Dupnik K., Schapiro J. M. and Shafer R. W.: Human Immunodeficiency Virus Reverse Transcriptase and Protease Sequence Database: an expanded data model integrating natural language text and sequence analysis programs. Nucleic Acids Res 2001; 29:296-9.

10. Boyd M., Duncombe C., Ruxrungthram K., Khongphattanayothin M., Hassink E., Srasuebkul P., Sangkote J., Reiss P., Stek M., Lange J., Cooper D. A. and Phanuphak P.: Indinavir TID vs Indinavir/Ritonavir BID in combination with AZT/3TC for HIV infection in nucleoside pretreated patients: HIV-NAT 005 76-week follow up. 9th Conference on Retroviruses and Opportunistisc Infections. Seattle, 2002.

11. Grabar S., Pradier C., Le Corfec E., Lancar R., Allavena C., Bentata M., Berlureau P., Dupont C., Fabbro-Peray P., Poizot-Martin I. and Costagliola D.: Factors associated with clinical and virological failure in patients receiving a triple therapy including a protease inhibitor. Aids 2000; 14:141-9.

12. Deeks S. G., Barbour J. D., Grant R. M. and Martin J. N.: Duration and predictors of CD4 T-cell gains in patients who continue combination therapy despite detectable plasma viremia. Aids 2002; 16:201-7.

13. Gazzard B. and Moyle G.: 1998 revision to the British HIV Association guidelines for antiretroviral treatment of HIV seropositive individuals. BHIVA Guidelines Writing Committee. Lancet 1998; 352:314-6.

14. Guidelines for the use of antiretroviral agents in HIV-infected adults and adolescents. Department of Health and Human Services and the Henry J. Kaiser Family Foundation. http://www.hivatis.org 2002.

15. Folkers G. and Morales J.: HIV treatment guidelines updated for adults and adolescents. http://www.hivatis.org. Accessed 2001.

16. Yeni P. G., Hammer S. M., Carpenter C. C., Cooper D. A., Fischl M. A., Gatell J. M., Gazzard B. G., Hirsch M. S., Jacobsen D. M., Katzenstein D. A., Montaner J. S., Richman D. D., Saag M. S., Schechter M., Schooley R. T., Thompson M. A., Vella S. and Volberding P. A.: Antiretroviral treatment for adult HIV infection in 2002: updated recommendations of the International AIDS Society-USA Panel. Jama 2002; 288:222-35.

17. Henry K.: The case for more cautious, patient-focused antiretroviral therapy. Ann Intern Med 2000; 132:306-11.

18. Palella F. J., Jr., Delaney K. M., Moorman A. C., Loveless M. O., Fuhrer J., Satten G. A., Aschman D. J. and Holmberg S. D.: Declining morbidity and mortality among patients with advanced human immunodeficiency virus infection. HIV Outpatient Study Investigators. N Engl J Med 1998; 338:853-60.

19. Egger M., May M., Chene G., Phillips A. N., Ledergerber B., Dabis F., Costagliola D., D'Arminio Monforte A., de Wolf F., Reiss P., Lundgren J. D., Justice A. C., Staszewski S., Leport C., Hogg R. S., Sabin C. A., Gill M. J., Salzberger B. and Sterne J. A.: Prognosis of HIV-1-infected patients starting highly active antiretroviral therapy: a collaborative analysis of prospective studies. Lancet 2002; 360:119-29.

20. Zhang L., Ramratnam B., Tenner-Racz K., He Y., Vesanen M., Lewin S., Talal A., Racz P., Perelson A. S., Korber B. T., Markowitz M. and Ho D. D.: Quantifying residual HIV-1 replication in patients receiving combination antiretroviral therapy. N Engl J Med 1999; 340:1605-13.

21. Katzenstein D. A., Hammer S. M., Hughes M. D., Gundacker H., Jackson J. B., Fiscus S., Rasheed S., Elbeik T., Reichman R., Japour A., Merigan T. C. and Hirsch M. S.: The relation of virologic and immunologic markers to clinical outcomes after nucleoside therapy in HIV-infected

adults with 200 to 500 CD4 cells per cubic millimeter. AIDS Clinical Trials Group Study 175 Virology Study Team. N Engl J Med 1996; 335:1091-8.

22. Autran B., Carcelain G., Li T. S., Blanc C., Mathez D., Tubiana R., Katlama C., Debre P. and Leibowitch J.: Positive effects of combined antiretroviral therapy on CD4+ T cell homeostasis and function in advanced HIV disease. Science 1997; 277:112-6.

23. Li T. S., Tubiana R., Katlama C., Calvez V., Ait Mohand H. and Autran B.: Long-lasting recovery in CD4 T-cell function and viral-load reduction after highly active antiretroviral therapy in advanced HIV-1 disease. Lancet 1998; 351:1682-6.

24. Wood E., Yip B., Hogg R. S., Sherlock C. H., Jahnke N., Harrigan R. P., O'Shaughnessy M. V. and Montaner J. S.: Full suppression of viral load is needed to achieve an optimal CD4 cell count response among patients on triple drug antiretroviral therapy. Aids 2000; 14:1955-60.

25. Plana M., Martinez C., Garcia F., Maleno M. J., Barcelo J. J., Garcia A., Lejeune M., Vidal C., Cruceta A., Miro J. M., Pumarola T., Gallart T. and Gatell J. M.: Immunologic reconstitution after 1 year of highly active antiretroviral therapy, with or without protease inhibitors. J Acquir Immune Defic Syndr 2002; 29:429-34.

26. John H., Muller N. J., Opravil M. and Hauri D.: Indinavir urinary stones as origin of upper urinary tract obstruction. Urol Int 1997; 59:257-9.

27. Henry K., Melroe H., Huebsch J., Hermundson J., Levine C., Swensen L. and Daley J.: Severe premature coronary artery disease with protease inhibitors. Lancet 1998; 351:1328.

28. Brinkman K., Smeitink J. A., Romijn J. A. and Reiss P.: Mitochondrial toxicity induced by nucleoside-analogue reverse- transcriptase inhibitors is a key factor in the pathogenesis of antiretroviral-therapy-related lipodystrophy. Lancet 1999; 354:1112-5.

29. Carr A., Samaras K., Thorisdottir A., Kaufmann G. R., Chisholm D. J. and Cooper D. A.: Diagnosis, prediction, and natural course of HIV-1 protease-inhibitor- associated lipodystrophy, hyperlipidaemia, and diabetes mellitus: a cohort study. Lancet 1999; 353:2093-9.

30. Carr A., Miller J., Law M. and Cooper D. A.: A syndrome of lipoatrophy, lactic acidaemia and liver dysfunction associated with HIV nucleoside analogue therapy: contribution to protease inhibitor-related lipodystrophy syndrome. Aids 2000; 14:F25-32.

31. Carr A.: Osteopenia in HIV infection. AIDS Clin Care 2001; 13:71-3, 8.

32. Paterson D. L., Swindells S., Mohr J., Brester M., Vergis E. N., Squier C., Wagener M. M. and Singh N.: Adherence to protease inhibitor therapy and outcomes in patients with HIV infection. Ann Intern Med 2000; 133:21-30.

33. Catz S. L., Kelly J. A., Bogart L. M., Benotsch E. G. and McAuliffe T. L.: Patterns, correlates, and barriers to medication adherence among persons prescribed new treatments for HIV disease. Health Psychol 2000; 19:124-33.

34. Bush C. E., Donovan R. M., Markowitz N., Baxa D., Kvale P. and Saravolatz L. D.: Gender is not a factor in serum human immunodeficiency virus type 1 RNA levels in patients with viremia. J Clin Microbiol 1996; 34:970-2.

35. Farzadegan H., Hoover D. R., Astemborski J., Lyles C. M., Margolick J. B., Markham R. B., Quinn T. C. and Vlahov D.: Sex differences in HIV-1 viral load and progression to AIDS. Lancet 1998; 352:1510-4.

36. Sterling T. R., Vlahov D., Astemborski J., Hoover D. R., Margolick J. B. and Quinn T. C.: Initial plasma HIV-1 RNA levels and progression to AIDS in women and men. N Engl J Med 2001; 344:720-5.

37. Staszewski S., Miller V., Sabin C., Schlecht C., Gute P., Stamm S., Leder T., Berger A., Weidemann E., Hill A. and Phillips A.: Determinants of sustainable CD4 lymphocyte count increases in response to antiretroviral therapy. Aids 1999; 13:951-6.

38. Lederman M. M. and Valdez H.: Immune restoration with antiretroviral therapies: implications for clinical management. Jama 2000; 284:223-8.

39. Thiebaut R., Morlat P., Jacqmin-Gadda H., Neau D., Mercie P., Dabis F. and Chene G.: Clinical progression of HIV-1 infection according to the viral response during the first year of antiretroviral treatment. Groupe d'Epidemiologie du SIDA en Aquitaine (GECSA). Aids 2000; 14:971-8.

40. Hogg R. S., Rhone S. A., Yip B., Sherlock C., Conway B., Schechter M. T., O'Shaughnessy M. V. and Montaner J. S.: Antiviral effect of double and triple drug combinations amongst HIV-infected adults: lessons from the implementation of viral load-driven antiretroviral therapy. Aids 1998; 12:279-84.

41. Palella Jr F. J., Chmiel J. S., Moorman A. C. and Holmberg S. D.: Durability and predictors of success of highly active antiretroviral therapy for ambulatory HIV-infected patients. Aids 2002; 16:1617-26.

42. Chene G., Binquet C., Moreau J. F., Neau D., Pellegrin I., Malvy D., Ceccaldi J., Lacoste D. and Dabis F.: Changes in CD4+ cell count and the risk of opportunistic infection or death after highly active antiretroviral treatment. Groupe d'Epidemiologie Clinique du SIDA en Aquitaine. Aids 1998; 12:2313-20.

43. McNaghten A. D., Hanson D. L., Jones J. L., Dworkin M. S. and Ward J. W.: Effects of antiretroviral therapy and opportunistic illness primary chemoprophylaxis on survival after AIDS diagnosis. Adult/Adolescent Spectrum of Disease Group. Aids 1999; 13:1687-95.

44. USPHS/IDSA Guidelines for the Prevention of Opportunistic Infections in Persons Infected with Human Immunodeficiency Virus November 2001 by the US Public Health Service (USPHS) and Infectious Diseases Society of America (IDSA); USPHS/IDSA Prevention of Opportunistic Infections Working Group.

45. Kaufmann D., Pantaleo G., Sudre P. and Telenti A.: CD4-cell count in HIV-1-infected individuals remaining viraemic with highly active antiretroviral therapy (HAART). Swiss HIV Cohort Study. Lancet 1998; 351:723-4.

46. Kaufmann G. R., Bloch M., Zaunders J. J., Smith D. and Cooper D. A.: Long-term immunological response in HIV-1-infected subjects receiving potent antiretroviral therapy. Aids 2000; 14:959-69.

47. Piketty C., Weiss L., Thomas F., Mohamed A. S., Belec L. and Kazatchkine M. D.: Long-term clinical outcome of human immunodeficiency virus-infected patients with discordant immunologic and virologic responses to a protease inhibitor-containing regimen. J Infect Dis 2001; 183:1328-35.

48. Garcia F., Vidal C., Plana M., Cruceta A., Gallart M. T., Pumarola T., Miro J. M. and Gatell J. M.: Residual low-level viral replication could explain discrepancies between viral load and CD4+ cell response in human immunodeficiency virus-infected patients receiving antiretroviral therapy. Clin Infect Dis 2000; 30:392-4.

49. Deeks S. G., Hecht F. M., Swanson M., Elbeik T., Loftus R., Cohen P. T. and Grant R. M.: HIV RNA and CD4 cell count response to protease inhibitor therapy in an urban AIDS clinic: response to both initial and salvage therapy. Aids 1999; 13:F35-43.

50. Lucas G. M., Chaisson R. E. and Moore R. D.: Highly active antiretroviral therapy in a large urban clinic: risk factors for virologic failure and adverse drug reactions. Ann Intern Med 1999; 131:81-7.

51. Walsh J. C., Hertogs K. and Gazzard B.: Viral drug resistance, adherence and pharmacokinetic indices in HIV-1 infected patients on successful and failing protease inhibitor based HAART. 40th Interscience Conference of Antimicrobial Agents and Chemotherapy. Canada, 2000

52. Montaner J. S., Reiss P., Cooper D., Vella S., Harris M., Conway B., Wainberg M. A., Smith D., Robinson P., Hall D., Myers M. and Lange J. M.: A randomized, double-blind trial comparing combinations of nevirapine, didanosine, and zidovudine for HIV-infected patients: the INCAS Trial. Italy, The Netherlands, Canada and Australia Study. Jama 1998; 279:930-7.

53. Arnsten J., Delmas P., Gourevitch M. and al. e.: Adherence and viral load in HIV-infected drug users: comparison of self-report and medication event monitors (MEMS). 7th Conference on Retroviruses and Opportunistic Infections. San Francisco, 2000

54. Havlir D. V., Hellmann N. S., Petropoulos C. J., Whitcomb J. M., Collier A. C., Hirsch M. S., Tebas P., Sommadossi J. P. and Richman D. D.: Drug susceptibility in HIV infection after viral rebound in patients receiving indinavir-containing regimens. Jama 2000; 283:229-34.

55. Shafer R. W., Winters M. A., Palmer S. and Merigan T. C.: Multiple concurrent reverse transcriptase and protease mutations and multidrug resistance of HIV-1 isolates from heavily treated patients. Ann Intern Med 1998; 128:906-11.

56. Hirsch M. S., Brun-Vezinet F., D'Aquila R. T., Hammer S. M., Johnson V. A., Kuritzkes D. R., Loveday C., Mellors J. W., Clotet B., Conway B., Demeter L. M., Vella S., Jacobsen D. M. and Richman D. D.: Antiretroviral drug resistance testing in adult HIV-1 infection: recommendations of an International AIDS Society-USA Panel. Jama 2000; 283:2417-26.

57. D'Aquila R. T., Schapiro J. M., Brun-Vezinet F., Clotet B., Conway B., Demeter L., Grant R. M., Johnson V. A., Kuritzkes D. R., Loveday C., Shafer R. W. and Richman D. D.: Drug resistance mutations in HIV-1. Topics in HIV medicine 2002; 10:11-5.

58. Meynard J.-L., Vray M., Morand-Joubert L., Race E., Descamps D., Peytavin G., Matheron S., Lamotte C., Guiramand S., Costagliola D., Brun-Vezinet F., Clavel F. and Girard P. M.: Phenotypic or genotypic resistance testing for choosing antiretroviral therapy after treatment failure: a randomized trial. AIDS 2002; 16:727-36.

59. Pieniazek D., Rayfield M., Hu D. J., Nkengasong J., Wiktor S. Z., Downing R., Biryahwaho B., Mastro T., Tanuri A., Soriano V., Lal R. and Dondero T.: Protease sequences from HIV-1 group M subtypes A-H reveal distinct amino acid mutation patterns associated with protease resistance in protease inhibitor-naive individuals worldwide. HIV Variant Working Group. Aids 2000; 14:1489-95.

60. Sato H., Tomita Y., Shibamura K., Shiino T., Miyakuni T. and Takebe Y.: Convergent evolution of reverse transcriptase (RT) genes of human immunodeficiency virus type 1 subtypes E and B following nucleoside analogue RT inhibitor therapies. J Virol 2000; 74:5357-62.

61. Vergne L., Peeters M., Mpoudi-Ngole E., Bourgeois A., Liegeois F., Toure-Kane C., Mboup S., Mulanga-Kabeya C., Saman E., Jourdan J., Reynes J. and Delaporte E.: Genetic diversity of protease and reverse transcriptase sequences in non-subtype-B human immunodeficiency virus type 1 strains: evidence of many minor drug resistance mutations in treatment-naive patients. J Clin Microbiol 2000; 38:3919-25.

62. Sirivichayakul S., Chantratita W., Sutthent R., Ruxrungtham K., Phanuphak P. and Oelrichs R. B.: Survey of reverse transcriptase from the heterosexual epidemic of human immunodeficiency virus type 1 CRF01_AE in Thailand from 1990 to 2000. AIDS Res Hum Retroviruses 2001; 17:1077-81.

63. Phanuphak P.: Potential antiretroviral drug resistance in developping countries: The Thailand Eperience. 9th Conference on Retroviruses and Opportunistic infections. Seattle, 2002.

64. Loveday C., Burke A., Van Hooff F. and Johnson M.: Equivalence in virological responses to HAART in patients with HIV-1 subtype non-B infections: a case-controlled study (n=100). 8th Retroconference on Retroviruses and Opportunistic Infections. Chicago, 2001.

65. Frater A. J., Dunn D. T., Beardall A. J., Ariyoshi K., Clarke J. R., McClure M. O. and Weber J. N.: Comparative response of African HIV-1-infected individuals to highly active antiretroviral therapy. Aids 2002; 16:1139-46.

66. Nijhuis M., Schuurman R., de Jong D., Erickson J., Gustchina E., Albert J., Schipper P., Gulnik S. and Boucher C. A.: Increased fitness of drug resistant HIV-1 protease as a result of acquisition of compensatory mutations during suboptimal therapy. Aids 1999; 13:2349-59.

67. Kaplan A. H., Michael S. F., Wehbie R. S., Knigge M. F., Paul D. A., Everitt L., Kempf D. J., Norbeck D. W., Erickson J. W. and Swanstrom R.: Selection of multiple human immunodeficiency virus type 1 variants that encode viral proteases with decreased sensitivity to an inhibitor of the viral protease. Proc Natl Acad Sci U S A 1994; 91:5597-601.

68. Martinez-Picado J., Savara A. V., Sutton L. and D'Aquila R. T.: Replicative fitness of protease inhibitor-resistant mutants of human immunodeficiency virus type 1. J Virol 1999; 73:3744-55.

69. Quinones-Mateu M. E., Ball S. C., Marozsan A. J., Torre V. S., Albright J. L., Vanham G., van Der Groen G., Colebunders R. L. and Arts E. J.: A dual infection/competition assay shows a correlation between ex vivo human immunodeficiency virus type 1 fitness and disease progression. J Virol 2000; 74:9222-33.

70. Zennou V., Mammano F., Paulous S., Mathez D. and Clavel F.: Loss of viral fitness associated with multiple Gag and Gag-Pol processing defects in human immunodeficiency virus type 1 variants selected for resistance to protease inhibitors in vivo. J Virol 1998; 72:3300-6.

71. McCune J. M., Namikawa R., Kaneshima H., Shultz L. D., Lieberman M. and Weissman I. L.: The SCID-hu mouse: murine model for the analysis of human hematolymphoid differentiation and function. Science 1988; 241:1632-9.

72. Glushakova S., Baibakov B., Zimmerberg J. and Margolis L. B.: Experimental HIV infection of human lymphoid tissue: correlation of CD4+ T cell depletion and virus syncytium-inducing/non-syncytium- inducing phenotype in histocultures inoculated with laboratory strains and patient isolates of HIV type 1. AIDS Res Hum Retroviruses 1997; 13:461-71.

73. Fischl M. A., Richman D. D., Grieco M. H., Gottlieb M. S., Volberding P. A., Laskin O. L., Leedom J. M., Groopman J. E., Mildvan D., Schooley R. T. and et al.: The efficacy of azidothymidine (AZT) in the treatment of patients with AIDS and AIDS-related complex. A double-blind, placebo-controlled trial. N Engl J Med 1987; 317:185-91.

74. Zidovudine, didanosine, and zalcitabine in the treatment of HIV infection: meta-analyses of the randomised evidence. HIV Trialists' Collaborative Group. Lancet 1999; 353:2014-25.

75. Gulick R. M., Mellors J. W., Havlir D., Eron J. J., Gonzalez C., McMahon D., Richman D. D., Valentine F. T., Jonas L., Meibohm A., Emini E. A. and Chodakewitz J. A.: Treatment with indinavir, zidovudine, and lamivudine in adults with human immunodeficiency virus infection and prior antiretroviral therapy. N Engl J Med 1997; 337:734-9.

76. Hammer S. M., Squires K. E., Hughes M. D., Grimes J. M., Demeter L. M., Currier J. S., Eron J. J., Jr., Feinberg J. E., Balfour H. H., Jr., Deyton L. R., Chodakewitz J. A. and Fischl M. A.: A controlled trial of two nucleoside analogues plus indinavir in persons with human immunodeficiency virus infection and CD4 cell counts of 200 per cubic millimeter or less. AIDS Clinical Trials Group 320 Study Team. N Engl J Med 1997; 337:725-33.

77. Cameron D. W., Heath-Chiozzi M., Danner S., Cohen C., Kravcik S., Maurath C., Sun E., Henry D., Rode R., Potthoff A. and Leonard J.: Randomised placebo-controlled trial of ritonavir in advanced HIV-1 disease. The Advanced HIV Disease Ritonavir Study Group. Lancet 1998; 351:543-9.

78. Delfraissy J. F.: Prise en charge therapeutique des personnes infectees par le VIH. 2000; :29-44.

79. Thursz M. R., Kwiatkowski D., Allsopp C. E., Greenwood B. M., Thomas H. C. and Hill A. V.: Association between an MHC class II allele and clearance of hepatitis B virus in the Gambia. N Engl J Med 1995; 332:1065-9.

80. Cramp M. E., Carucci P., Underhill J., Naoumov N. V., Williams R. and Donaldson P. T.: Association between HLA class II genotype and spontaneous clearance of hepatitis C viraemia. J Hepatol 1998; 29:207-13.

81. Minton E. J., Smillie D., Neal K. R., Irving W. L., Underwood J. C. and James V.: Association between MHC class II alleles and clearance of circulating hepatitis C virus. Members of the Trent Hepatitis C Virus Study Group. J Infect Dis 1998; 178:39-44.

82. Ezekowitz A.: Mannose-binding protein and susceptibility to chronic hepatitis B infection. Lancet 1996; 348:1396-7.

83. Garred P., Madsen H. O., Balslev U., Hofmann B., Pedersen C., Gerstoft J. and Svejgaard A.: Susceptibility to HIV infection and progression of AIDS in relation to variant alleles of mannose-binding lectin. Lancet 1997; 349:236-40.

84. Carrington M., Nelson G. W., Martin M. P., Kissner T., Vlahov D., Goedert J. J., Kaslow R., Buchbinder S., Hoots K. and O'Brien S. J.: HLA and HIV-1: heterozygote advantage and B*35-Cw*04 disadvantage. Science 1999; 283:1748-52.

85. Deng H., Liu R., Ellmeier W., Choe S., Unutmaz D., Burkhart M., Di Marzio P., Marmon S., Sutton R. E., Hill C. M., Davis C. B., Peiper S. C., Schall T. J., Littman D. R. and Landau N. R.: Identification of a major co-receptor for primary isolates of HIV-1. Nature 1996; 381:661-6.

86. Dragic T., Litwin V., Allaway G. P., Martin S. R., Huang Y., Nagashima K. A., Cayanan C., Maddon P. J., Koup R. A., Moore J. P. and Paxton W. A.: HIV-1 entry into CD4+ cells is mediated by the chemokine receptor CC- CKR-5. Nature 1996; 381:667-73.

87. Alkhatib G., Combadiere C., Broder C. C., Feng Y., Kennedy P. E., Murphy P. M. and Berger E. A.: CC CKR5: a RANTES, MIP-1alpha, MIP-1beta receptor as a fusion cofactor for macrophage-tropic HIV-1. Science 1996; 272:1955-8.

88. Samson M., Libert F., Doranz B. J., Rucker J., Liesnard C., Farber C. M., Saragosti S., Lapoumeroulie C., Cognaux J., Forceille C., Muyldermans G., Verhofstede C., Burtonboy G., Georges M., Imai T., Rana S., Yi Y., Smyth R. J., Collman R. G., Doms R. W., Vassart G. and Parmentier M.: Resistance to HIV-1 infection in caucasian individuals bearing mutant alleles of the CCR-5 chemokine receptor gene. Nature 1996; 382:722-5.

89. Martin M. P., Dean M., Smith M. W., Winkler C., Gerrard B., Michael N. L., Lee B., Doms R. W., Margolick J., Buchbinder S., Goedert J. J., O'Brien T. R., Hilgartner M. W., Vlahov D., O'Brien

S. J. and Carrington M.: Genetic acceleration of AIDS progression by a promoter variant of CCR5. Science 1998; 282:1907-11.

90. McDermott D. H., Zimmerman P. A., Guignard F., Kleeberger C. A., Leitman S. F. and Murphy P. M.: CCR5 promoter polymorphism and HIV-1 disease progression. Multicenter AIDS Cohort Study (MACS). Lancet 1998; 352:866-70.

91. Kostrikis L. G., Huang Y., Moore J. P., Wolinsky S. M., Zhang L., Guo Y., Deutsch L., Phair J., Neumann A. U. and Ho D. D.: A chemokine receptor CCR2 allele delays HIV-1 disease progression and is associated with a CCR5 promoter mutation. Nat Med 1998; 4:350-3.

92. Kostrikis L. G.: Impact of natural chemokine receptor polymorphisms on perinatal transmission of human immunodeficiency virus type 1. Teratology 2000; 61:387-90.

93. Liu H., Shioda T., Nagai Y., Iwamoto A., Wasi C., Ma J., Liang W., Takebe Y., Theodorou I., Magierowska M., Krishnamoorty R., Chaventre A. and Debre P.: Distribution of HIV-1 disease modifying regulated on activation normal T cell expressed and secreted haplotypes in Asian, African and Caucasian individuals. French ALT and IMMUNOCO Study Group. Aids 1999; 13:2602-3.

94. Liu H., Chao D., Nakayama E. E., Taguchi H., Goto M., Xin X., Takamatsu J. K., Saito H., Ishikawa Y., Akaza T., Juji T., Takebe Y., Ohishi T., Fukutake K., Maruyama Y., Yashiki S., Sonoda S., Nakamura T., Nagai Y., Iwamoto A. and Shioda T.: Polymorphism in RANTES chemokine promoter affects HIV-1 disease progression. Proc Natl Acad Sci U S A 1999; 96:4581-5.

95. Faure S., Meyer L., Costagliola D., Vaneensberghe C., Genin E., Autran B., Delfraissy J. F., McDermott D. H., Murphy P. M., Debre P., Theodorou I. and Combadiere C.: Rapid progression to AIDS in HIV+ individuals with a structural variant of the chemokine receptor CX3CR1. Science 2000; 287:2274-7.

96. McDermott D. H., Colla J. S., Kleeberger C. A., Plankey M., Rosenberg P. S., Smith E. D., Zimmerman P. A., Combadiere C., Leitman S. F., Kaslow R. A., Goedert J. J., Berger E. A., O'Brien T. R. and Murphy P. M.: Genetic polymorphism in CX3CR1 and risk of HIV disease. Science 2000; 290:2031.

97. Nakayama E. E., Hoshino Y., Xin X., Liu H., Goto M., Watanabe N., Taguchi H., Hitani A., Kawana-Tachikawa A., Fukushima M., Yamada K., Sugiura W., Oka S. I., Ajisawa A., Sato H., Takebe Y., Nakamura T., Nagai Y., Iwamoto A. and Shioda T.: Polymorphism in the interleukin-4 promoter affects acquisition of human immunodeficiency virus type 1 syncytium-inducing phenotype. J Virol 2000; 74:5452-9.

98. Shin H. D., Winkler C., Stephens J. C., Bream J., Young H., Goedert J. J., O'Brien T. R., Vlahov D., Buchbinder S., Giorgi J., Rinaldo C., Donfield S., Willoughby A., O'Brien S. J. and Smith M. W.: Genetic restriction of HIV-1 pathogenesis to AIDS by promoter alleles of IL10. Proc Natl Acad Sci U S A 2000; 97:14467-72.

99. Fellay J., Marzolini C., Meaden E. R., Back D. J., Buclin T., Chave J. P., Decosterd L. A., Furrer H., Opravil M., Pantaleo G., Retelska D., Ruiz L., Schinkel A. H., Vernazza P., Eap C. B. and Telenti A.: Response to antiretroviral treatment in HIV-1-infected individuals with allelic variants of the multidrug resistance transporter 1: a pharmacogenetics study. Lancet 2002; 359:30-6.

100. Ameyaw M. M., Regateiro F., Li T., Liu X., Tariq M., Mobarek A., Thornton N., Folayan G. O., Githang'a J., Indalo A., Ofori-Adjei D., Price-Evans D. A. and McLeod H. L.: MDR1 pharmacogenetics: frequency of the C3435T mutation in exon 26 is significantly influenced by ethnicity. Pharmacogenetics 2001; 11:217-21.

101. Xie H. G., Kim R. B., Wood A. J. and Stein C. M.: Molecular basis of ethnic differences in drug disposition and response. Annu Rev Pharmacol Toxicol 2001; 41:815-50.

102. Mallal S., Nolan D., Witt C., Masel G., Martin A. M., Moore C., Sayer D., Castley A., Mamotte C., Maxwell D., James I. and Christiansen F. T.: Association between presence of HLA-B*5701, HLA-DR7, and HLA-DQ3 and hypersensitivity to HIV-1 reverse-transcriptase inhibitor abacavir. Lancet 2002; 359:727-32.

103. Weniger B. G., Limpakarnjanarat K., Ungchusak K., Thanprasertsuk S., Choopanya K., Vanichseni S., Uneklabh T., Thongcharoen P. and Wasi C.: The epidemiology of HIV infection and AIDS in Thailand. Aids 1991; 5:S71-85.

104. WHO: HIV/AIDS in Asia and the Pacific Region. 2001.

105. Sutthent R., Foongladda S., Chearskul S., Wanaprapa N., Likanontsakul S., Kositanont U., Riengrojpitak S., Sahapong S. and Wasi C.: Maternal and viral factors in vertical transmission of HIV-1 subtype E. Southeast Asian J Trop Med Pub Health 1997; 28:689-97.

106. Sutthent R., Foongladda S., Chearskul S., Wanprapa N., Likanonskul S., Kositanont U., Riengrojpitak S., Sahaphong S. and Wasi C.: V3 sequence diversity of HIV-1 subtype E in infected mothers and their infants. J Acquir Immune Defic Syndr Hum Retrovirol 1998; 18:323-31.

107. Sriinsut A., Young N. L., Chaowanachan T., Wasinrapee P., Kaewpunt N., Suksrisupanich O., Wacharapluesadee S., Leelawiwat W., Pau C. P., Limpalarnjanarat K. and Mastro T. D.: V3-loop peptide serology for determination of HIV-1 subtypes in Thailand. Fifth Intl Cong on AIDS in Asia and the Pacific,. Kuala Lumpur, 1999

108. Nelson K. E., Rungruengthanakit K., Margolick J., Suriyanon V., Niyomthai S., de Boer M. A., Kawichai S., Robison V., Celentano D. D., Nagachinta T. and Duerr A.: High rates of transmission of subtype E human immunodeficiency virus type 1 among heterosexual couples in Northern Thailand: role of sexually transmitted diseases and immune compromise. J Infect Dis 1999; 180:337-43.

109. Berman J.: Thailand attacks AIDS with two-pronged approach. Lancet 1999; 353:1600.

110. Thaineua V., Sirinirund P., Tanbanjong A., Lallemant M., Soucat A. and Lamboray J. L.: From research to practice: use of short course zidovudine to prevent mother-to-child HIV transmission in the context of routine health care in Northern Thailand. Southeast Asian J Trop Med Public Health 1998; 29:429-42.

111. World Bank: Thailand's response to AIDS: building on success, confronting the future, 2001.

112. Surasiengsunk S., Kiranandana S., Wongboonsin K., Garnett G. P., Anderson R. M. and van Griensven G. J.: Demographic impact of the HIV epidemic in Thailand. Aids 1998; 12:775-84.

113.Unicef EAPRO: Estimating the numbers of children affected, Mekong Children and HIV/AIDS, 2000.

114. Connor E. M., Sperling R. S., Gelber R., Kiselev P., Scott G., O'Sullivan M. J., VanDyke R., Bey M., Shearer W., Jacobson R. L. and et al.: Reduction of maternal-infant transmission of human immunodeficiency virus type 1 with zidovudine treatment. Pediatric AIDS Clinical Trials Group Protocol 076 Study Group. N Engl J Med 1994; 331:1173-80.

115. Lallemant M., Jourdain G., Le Coeur S., Kim S., Koetsawang S., Comeau A. M., Phoolcharoen W., Essex M., McIntosh K., Vithayasai V. and Investigators. f. t. P. H. P. T. T.: A trial of shortened zidovudine regimens to prevent mother-to-child transmission of Human Immunodeficiency Virus 1. N Engl J Med 2000; 343:982-91.

116. Guay L. A., Musoke P., Fleming T., Bagenda D., Allen M., Nakabiito C., Sherman J., Bakaki P., Ducar C., Deseyve M., Emel L., Mirochnick M., Fowler M. G., Mofenson L., Miotti P., Dransfield K., Bray D., Mmiro F. and Jackson J. B.: Intrapartum and neonatal single-dose nevirapine compared with zidovudine for prevention of mother-to-child transmission of HIV-1 in Kampala, Uganda: HIVNET 012 randomised trial. Lancet 1999; 354:795-802.

117. Lallemant M., Thaineua V., Jourdain G., Le Coeur S., Koetsawang S., Kosalaraksa P., Mary J. Y. and Ngo N.: PHPT-2: Nevirapine Plus Zidovudine For The Prevention Of Perinatal HIV. XIV International AIDS Conference. Barcelona, 2002.

118. Jourdain G., Tod M., Le Coeur S., Schaffer V., Wattanaporn P., Hemvuttiphan J. and Lallemant M.: Pharmacokinetics of Zidovudine (ZDV) during labor (Thailand). 5th International Congress on AIDS in Asia and the Pacific. Kuala-lumpur, Indonesia, 1999

119. Condra J. H., Schleif W. A., Blahy O. M., Gabryelski L. J., Graham D. J., Quintero J. C., Rhodes A., Robbins H. L., Roth E., Shivaprakash M. and et al.: In vivo emergence of HIV-1 variants resistant to multiple protease inhibitors. Nature 1995; 374:569-71.

120. Molla A., Korneyeva M., Gao Q., Vasavanonda S., Schipper P. J., Mo H. M., Markowitz M., Chernyavskiy T., Niu P., Lyons N., Hsu A., Granneman G. R., Ho D. D., Boucher C. A., Leonard J. M., Norbeck D. W. and Kempf D. J.: Ordered accumulation of mutations in HIV protease confers resistance to ritonavir. Nat Med 1996; 2:760-6.

121. Bartlett J. A., DeMasi R., Quinn J., Moxham C. and Rousseau F.: Overview of the effectiveness of triple combination therapy in antiretroviral-naive HIV-1 infected adults. Aids 2001; 15:1369-77.

122. Baxter J. D., Mayers D. L., Wentworth D. N., Neaton J. D., Hoover M. L., Winters M. A., Mannheimer S. B., Thompson M. A., Abrams D. I., Brizz B. J., Ioannidis J. P. and Merigan T. C.: A randomized study of antiretroviral management based on plasma genotypic antiretroviral resistance testing in patients failing therapy. CPCRA 046 Study Team for the Terry Beirn Community Programs for Clinical Research on AIDS. Aids 2000; 14:F83-93.

123. Durant J., Clevenbergh P., Halfon P., Delgiudice P., Porsin S., Simonet P., Montagne N., Boucher C. A., Schapiro J. M. and Dellamonica P.: Drug-resistance genotyping in HIV-1 therapy: the VIRADAPT randomised controlled trial. Lancet 1999; 353:2195-9.

124. Ledergerber B., Egger M., Opravil M., Telenti A., Hirschel B., Battegay M., Vernazza P., Sudre P., Flepp M., Furrer H., Francioli P. and Weber R.: Clinical progression and virological failure on highly active antiretroviral therapy in HIV-1 patients: a prospective cohort study. Swiss HIV Cohort Study. Lancet 1999; 353:863-8.

125. Faye A., Race E., Obry V., Prevot M. H., Joly V., Matheron S., Damond F., Dam E., Paulous S. and Clavel F.: Viral fitness in patients with discordant CD4 and plasma HIV RNA evolution following protease inhibitor failure. 6th Conference on Retroviruses and Opportunistic Infections. Chicago (IL), 1999.

126. Stoddart C., Mammano F., Moreno M., Linquist-Stepps V., Bare C., Clavel F. and McCune J. M.: Lack of fitness of protease inhibitor-resistant HIV-1 in vivo. 6th Conference on Retroviruses and Opportunistic Infections. Chicago (IL), 1999.

127. d'Arminio Monforte A., Testori V., Adorni F., Castelnuovo B., Bini T., Testa L., Moscatelli G., Chiesa E., Rusconi S., Abeli C., Sollima S., Musicco M., Meroni L., Galli M. and Moroni M.: CD4 cell counts at the third month of HAART may predict clinical failure. Aids 1999; 13:1669-76.

128. Coakley E. P., Samore M. H., Gillis J. M., Hughes M. D. and Hammer S. M.: The values of quantitative serum HIV-1 RNA levels and CD4 cell counts for predicting survival time among HIV-positive individuals with CD4 counts of or = 50 x 10(6) cells/l. Aids 2000; 14:1147-53.

129. Hogg R. S., Yip B., Chan K. J., Wood E., Craib K. J., O'Shaughnessy M. V. and Montaner J. S.: Rates of Disease Progression by Baseline CD4 Cell Count and Viral Load After Initiating Triple-Drug Therapy. Jama 2001; 286:2568-77.

130. Sterling T. R., Chaisson R. E. and Moore R. D.: HIV-1 RNA, CD4 T-lymphocytes, and clinical response to highly active antiretroviral therapy. Aids 2001; 15:2251-7.

131. Marschner I. C., Collier A. C., Coombs R. W., D'Aquila R. T., DeGruttola V., Fischl M. A., Hammer S. M., Hughes M. D., Johnson V. A., Katzenstein D. A., Richman D. D., Smeaton L. M., Spector S. A. and Saag M. S.: Use of changes in plasma levels of human immunodeficiency virus type 1 RNA to assess the clinical benefit of antiretroviral therapy. J Infect Dis 1998; 177:40-7.

132. Grabar S., Le Moing V., Goujard C., Leport C., Kazatchkine M. D., Costagliola D. and Weiss L.: Clinical outcome of patients with HIV-1 infection according to immunologic and virologic response after 6 months of highly active antiretroviral therapy. Ann Intern Med 2000; 133:401-10.

133. Abgrall S., Duval X., Joly V., Descamps D., Matheron S. and Costagliola D.: Mid-term clinical and immunologic outcome of HIV infection according to virologic efficacy during the year following initial virus undetectability of HAART. submitted.

134. Deeks S. G., Wrin T., Liegler T., Hoh R., Hayden M., Barbour J. D., Hellmann N. S., Petropoulos C. J., McCune J. M., Hellerstein M. K. and Grant R. M.: Virologic and immunologic consequences of discontinuing combination antiretroviral-drug therapy in HIV-infected patients with detectable viremia. N Engl J Med 2001; 344:472-80.

135. Staszewski S., Morales-Ramirez J., Tashima K. T., Rachlis A., Skiest D., Stanford J., Stryker R., Johnson P., Labriola D. F., Farina D., Manion D. J. and Ruiz N. M.: Efavirenz plus zidovudine and lamivudine, efavirenz plus indinavir, and indinavir plus zidovudine and lamivudine in the treatment of HIV-1 infection in adults. Study 006 Team. N Engl J Med 1999; 341:1865-73.

136. Mocroft A., Devereux H., Kinloech-de-Loes S., Wilson D., Madge S., Youle M., Tyrer M., Loveday C., Phillips A. N. and Johnson M. A.: Immunological , virological and clinical response to highly active antiretroviral therapy treatment regimens in a complete clinic population. AIDS 2000; 14:1545-52.

137. Mocroft A., Youle M., Moore A., Sabin C. A., Madge S., Lepri A. C., Tyrer M., Chaloner C., Wilson D., Loveday C., Johnson M. A. and Phillips A. N.: Reasons for modification and discontinuation of antiretrovirals: results from a single treatment centre. Aids 2001; 15:185-94.

138. d'Arminio Monforte A., Lepri A. C., Rezza G., Pezzotti P., Antinori A., Phillips A. N., Angarano G., Colangeli V., De Luca A., Ippolito G., Caggese L., Soscia F., Filice G., Gritti F., Narciso P., Tirelli U. and Moroni M.: Insights into the reasons for discontinuation of the first highly active antiretroviral therapy (HAART) regimen in a cohort of antiretroviral naive patients. I.CO.N.A. Study Group. Italian Cohort of Antiretroviral-Naive Patients. Aids 2000; 14:499-507.

139. Holder D. J., Condra J. H., Schleif W. A., Chodakewitz J. and Emini E. A.: Virologic failure during combination therapy with Crixivan and RT inhibitors is often associated with expression of resistance-associated mutations in RT only. 6th Conference on Retroviruses and Opportunistic Infections,. Chicago, IL., 1999

140. Descamps D., Flandre P., Calvez V., Peytavin G., Meiffredy V., Collin G., Delaugerre C., Robert-Delmas S., Bazin B., Aboulker J. P., Pialoux G., Raffi F. and Brun-Vezinet F.: Mechanisms of virologic failure in previously untreated HIV-infected patients from a trial of induction-maintenance therapy. Trilege (Agence Nationale de Recherches sur le SIDA 072) Study Team). Jama 2000; 283:205-11.

141. Harries A. D., Nyangulu D. S., Hargreaves N. J., Kaluwa O. and Salaniponi F. M.: Preventing antiretroviral anarchy in sub-Saharan Africa. Lancet 2001; 358:410-4.

142. WHO initiative on HIV/AIDS and STI: Safe and effective use of antiretroviral treatment in adults with particular references to resource limited settings, 2000.

143. Farmer P., Leandre F., Mukherjee J. S., Claude M., Nevil P., Smith-Fawzi M. C., Koenig S. P., Castro A., Becerra M. C., Sachs J., Attaran A. and Kim J. Y.: Community-based approaches to HIV treatment in resource-poor settings. Lancet 2001; 358:404-9.

144. Weidle P. J., Malamba S., Mwebaze R., Sozi C., Rukundo G., Downing R., Hanson D., Ochola D., Mugyenyi P., Mermin J., Samb B. and Lackritz E.: Assessment of a pilot antiretroviral drug therapy programme in Uganda: patients' response, survival, and drug resistance. Lancet 2002; 360:34-40.

145. Koetsawang S.: Evaluation of counseling in the Perinatal HIV Prevention Trial in Thailand. 5th International Conference on AIDS in Asia and Pacific, Sat. Symposium on PMTCT. Kuala Lumpur, Malaysia, 1999

146. Connor E. M. and Mofenson L. M.: Zidovudine for the reduction of perinatal human immunodeficiency virus transmission: pediatric AIDS Clinical Trials Group Protocol 076-- results and treatment recommendations. Pediatr Infect Dis J 1995; 14:536-41.

147. Wong J. K., Gunthard H. F., Havlir D. V., Zhang Z. Q., Haase A. T., Ignacio C. C., Kwok S., Emini E. and Richman D. D.: Reduction of HIV-1 in blood and lymph nodes following potent antiretroviral therapy and the virologic correlates of treatment failure. Proc Natl Acad Sci U S A 1997; 94:12574-9.

148. Hogg R. S., Yip B., Kully C., Craib K. J., O'Shaughnessy M. V., Schechter M. T. and Montaner J. S.: Improved survival among HIV-infected patients after initiation of triple-drug antiretroviral regimens. Cmaj 1999; 160:659-65.

149. Gulick R. M., Mellors J. W., Havlir D., Eron J. J., Meibohm A., Condra J. H., Valentine F. T., McMahon D., Gonzalez C., Jonas L., Emini E. A., Chodakewitz J. A., Isaacs R. and Richman D. D.: 3-year suppression of HIV viremia with indinavir, zidovudine, and lamivudine. Ann Intern Med 2000; 133:35-9.

150. National Guidelines for the clinical management of HIV infection in children and adult, Ministry of Public Health,Thailand, 2000.

151. Farmer K. C.: Methods for measuring and monitoring medication regimen adherence in clinical trials and clinical practice. Clin Ther 1999; 21:1074-90; discussion 3.

152. Haubrich R. H., Little S. J., Currier J. S., Forthal D. N., Kemper C. A., Beall G. N., Johnson D., Dube M. P., Hwang J. Y. and McCutchan J. A.: The value of patient-reported adherence to antiretroviral therapy in predicting virologic and immunologic response. California Collaborative Treatment Group. Aids 1999; 13:1099-107.

153. Moatti J. P., Carrieri M. P., Spire B., Gastaut J. A., Cassuto J. P. and Moreau J.: Adherence to HAART in French HIV-infected injecting drug users: the contribution of buprenorphine drug maintenance treatment. The Manif 2000 study group. Aids 2000; 14:151-5.

154. Chesney M. A., Ickovics J. R., Chambers D. B., Gifford A. L., Neidig J., Zwickl B. and Wu A. W.: Self-reported adherence to antiretroviral medications among participants in HIV clinical trials: the AACTG adherence instruments. Patient Care Committee & Adherence Working Group of the Outcomes Committee of the Adult AIDS Clinical Trials Group (AACTG). AIDS Care 2000; 12:255-66.

155. Ho C. F., Fong O. W. and Wong K. H.: Patient self-report as a marker of adherence to antiretroviral therapy. Clin Infect Dis 2002; 34:1534-5.

156. Sollitto S., Mehlman M., Youngner S. and Lederman M. M.: Should physicians withhold highly active antiretroviral therapies from HIV-AIDS patients who are thought to be poorly adherent to treatment? Aids 2001; 15:153-9.

157. Boucher C. A., Lange J. M., Miedema F. F., Weverling G. J., Koot M., Mulder J. W., Goudsmit J., Kellam P., Larder B. A. and Tersmette M.: HIV-1 biological phenotype and the development of zidovudine resistance in relation to disease progression in asymptomatic individuals during treatment. Aids 1992; 6:1259-64.

158. International conference on harmonisation of technical requirements for registration of pharmaceuticals for human use. Good clinical practice: consolidated guideline: availability. Fed regist 1997; 62:25691-709.

159. Blackwelder W. C.: "Proving the null hypothesis" in clinical trials. Control Clin Trials 1982; 3:345-53.

160. Dunnett C. W. and Gent M.: Significance testing to establish equivalence between treatments with special reference to data in the form of 2x2 tables. Biometrics 1977; 33:593-602.

161. Junghans C., Low N., Chan P., Witschi A., Vernazza P. and Egger M.: Uniform risk of clinical progression despite differences in utilization of highly active antiretroviral therapy: Swiss HIV Cohort Study. Aids 1999; 13:2547-54.

162. O'Brien P. C. and Fleming T. R.: A multiple testing procedure for clinical trials. Biometrics 1997; 35:549-56.

163. Lan K. K. G. and DeMets D. L.: Discrete sequential boundaries for clinical trials. Biometrica 1983; 70:659-63.

164. Agresti A.: Analysis of ordinal categorical data, JohnWiley and Sons, 1984, p 275

165. Makuch R. and Simon R.: Sample size requirements for evaluating a conservative therapy. Cancer Treat Rep 1978; 62:1037-40.

166. Barry M., Wild M., Veal G., Back D., Breckenridge A., Fox R., Beeching N., Nye F., Carey P. and Timmins D.: Zidovudine phosphorylation in HIV-infected patients and seronegative volunteers. Aids 1994; 8:F1-5.

167. Wattanagoon Y., Na Bangchang K., Hoggard P. G., Khoo S. H., Gibbons S. E., Phiboonbhanakit D., Karbwang J. and Back D. J.: Pharmacokinetics of zidovudine phosphorylation in human immunodeficiency virus-positive thai patients and healthy volunteers. Antimicrob Agents Chemother 2000; 44:1986-9.

168. Ungsedhapand C., Kroon E. D., Suwanagool S., Ruxrungtham K., Yimsuan N., Sonjai A., Ubolyam S., Buranapraditkun S., Tiengrim S., Pakker N., Kunanusont C., Lange J. M., Cooper D. A. and Phanuphak P.: A randomized, open-label, comparative trial of zidovudine plus lamivudine versus zidovudine plus lamivudine plus didanosine in antiretroviral-naive HIV-1-infected Thai patients. J Acquir Immune Defic Syndr 2001; 27:116-23.

169. Hsu A., Granneman G. R., Cao G., Carothers L., Japour A., El-Shourbagy T., Dennis S., Berg J., Erdman K., Leonard J. M. and Sun E.: Pharmacokinetic interaction between ritonavir and indinavir in healthy volunteers. Antimicrob Agents Chemother 1998; 42:2784-91.

170. Burger D., Felderhof M., Panuphak P., Duncombe C., Mahanontharit A., Yeamwanichnun W., Ubolyam S., Cooper D., Stek M., Lange J. and Reiss P.: Both short-term virological efficacy and drug-associated nephrotoxicity are related to indinavir (IDV) pharmacokinetics (PK) in HIV-1 infected Thai patients. 8th Conference on Retroviruses and Opportunistisc Infections. Chicago, 2001.

171. Podzamczer D., Arrizabalaga J., Van Wanzeele P., Harris M., Pedersen C., Cahn P., Casiro A., Chiodo F., Arnaiz J. A., Gatell J. M. and Team f. t. B. S.: Continued indinavir (800 mg tid) versus switching to indinavir + ritonavir (800/100mg bid) in HIV patients having achieved viral load suppression. A randomized study: the BID efficacy and safety trial (BEST). Aids 2000; 14:S8.

172. Saah A. J., Winchell G. A., Nessly M. L., Seniuk M. A., Rhodes R. R. and Deutsch P. J.: Pharmacokinetic profile and tolerability of indinavir-ritonavir combinations in healthy volunteers. Antimicrob Agents Chemother 2001; 45:2710-5.

173. Katlama C., Lamotte C., Hait Mohand H., Calvez V., Agher R., Bricaire F. and Peytavin G.: Ritonavir (RTV)/Indinavir (IDV) (100/400 mg BID): a simple, effective and well tolerated PI containing regimen. 1st IAS Conference on HIV pathogenesis and treatment. Buenos Aires, Argentina, 2001.
